# Machine learning prediction models for deep vein thrombosis in hospitalized patients: a systematic review and meta-analysis

**DOI:** 10.3389/fmed.2026.1849096

**Published:** 2026-05-26

**Authors:** An Liu, Kasturi Dewi Varathan, Vimala Ramoo, Thalwaththe Gedara Nadeeka Shayamalie Gunarathne

**Affiliations:** 1 Department of Nursing Science, Faculty of Medicine, Universiti Malaya, Kuala Lumpur, Malaysia; 2 Department of Information System, Faculty of Computer Science and Information Technology, Universiti Malaya, Kuala Lumpur, Malaysia

**Keywords:** deep vein thrombosis, machine learning, meta-analysis, prediction model, systematic review

## Abstract

**Objectives:**

To systematically review machine learning–based prediction models for deep vein thrombosis (DVT) in hospitalized patients and to evaluate their methodological quality, predictive performance, and clinical applicability.

**Methods:**

This systematic review was conducted in accordance with PRISMA and registered in PROSPERO (CRD420251069584). PubMed, Embase, Web of Science, and CINAHL were searched from November 1, 2015, to November 1, 2025. Studies developing, validating, or updating machine learning–based prediction models for DVT in hospitalized adults were included. Data were extracted on study characteristics, data sources, predictors, modeling methods, validation strategies, and model performance. Risk of bias and applicability were assessed using PROBAST. A meta-analysis of validation AUCs was performed in R, and heterogeneity was explored using the Cochrane Q test, I^2^ statistic, subgroup analysis, and sensitivity analysis.

**Results:**

A total of 983 studies were initially identified through database searches. Following screening, 17 studies met the inclusion criteria, of which 10 were included in the meta-analysis. D-dimer, surgery, and age emerged as the most frequently reported predictors across models. Six studies were judged to be at high risk of bias, mainly because of limitations in the analysis domain, including inadequate reporting of missing-data handling, calibration, and validation procedures. The pooled AUC of the 10 validated models was 0.85 (95% CI: 0.81–0.90), indicating good overall discrimination; however, heterogeneity was substantial and remained high in subgroup and sensitivity analyses.

**Conclusion:**

Machine learning–based models for predicting DVT in hospitalized patients show promising discriminative performance, but their clinical applicability remains limited by methodological weaknesses, poor reporting, substantial heterogeneity, and insufficient external validation. Future studies should prioritize multicenter external validation, transparent reporting, and head-to-head comparison with established clinical risk assessment tools.

## 1 Introduction

Deep vein thrombosis (DVT) is a common complication among hospitalized patients, with reported incidence ranging from 1 to 37.77% across different clinical conditions (1). DVT is the major source of pulmonary embolism (PE), one of the most serious and potentially fatal complications of hospital-associated venous thromboembolism (2–4). Therefore, early identification of patients at high risk of DVT is essential for implementing timely and targeted preventive or therapeutic interventions (5).

Currently, venous thromboembolism risk assessment in clinical practice still relies largely on conventional paper-based scoring tools (6). However, these tools often show limited predictive performance in heterogeneous inpatient populations, such as patients in intensive care units, postoperative patients, and patients with cancer (7). In addition, some laboratory indicators and clinical variables included in these scales are not readily available in routine practice, which may further compromise predictive accuracy (8). Moreover, most existing scales were developed in Western populations, which may limit their generalizability to patients from other geographical and clinical settings (9).

With the growing availability of electronic medical records and routinely collected clinical data, machine learning has become increasingly important in the development of clinical prediction models (10). Compared with conventional statistical approaches, machine learning methods can better accommodate high-dimensional data, model complex nonlinear relationships, and detect interactions among multiple predictors (11). This is particularly relevant to DVT in hospitalized patients, where risk is shaped by a combination of demographic factors, comorbidities, laboratory findings, treatment-related exposures, and disease-specific conditions (12). As a result, machine-learning-based prediction models are being developed with increasing frequency to support DVT risk assessment in hospitalized patients.

Despite this growing interest, the methodological quality, predictive performance, and clinical applicability of these models have not yet been systematically synthesized. Therefore, this study aimed to systematically review published prediction models for DVT in hospitalized patients. The findings of this review may help guide future model development, external validation, and clinical implementation.

## 2 Methods

The study protocol was registered on PROSPERO (registration number: CRD420251069584).

### 2.1 Search strategy

A comprehensive and systematic literature search was conducted in PubMed, Embase, Web of Science, and the Cumulative Index to Nursing and Allied Health Literature (CINAHL) from November 1, 2015 to November 1, 2025. Searches were limited to English-language publications. The search strategy combined controlled vocabulary terms (e.g., MeSH and Emtree) and free-text keywords related to: (1) machine learning and prediction modeling (e.g., “machine learning,” “ML,” “predictive modeling,” and “clinical prediction models”); and (2) deep vein thrombosis (DVT; e.g., “phlebothrombosis” and “lower extremity deep vein thrombosis”). Boolean operators (AND, OR) were used to combine these terms. Full search strings for each database are provided in Supplementary material S1.

### 2.2 Inclusion and exclusion criteria

This review followed the Preferred Reporting Items for Systematic Reviews and Meta-Analyses (PRISMA) guidelines. Studies were eligible if they met the following criteria: (1) included hospitalized adult patients aged 18 years or older; (2) used an observational study design; (3) defined deep vein thrombosis as the primary outcome or as part of the target outcome; (4) developed, validated, or updated a clinical prediction model; and (5) were published in English. Studies were excluded if they (1) examined risk factors only without developing a prediction model; (2) did not describe the model development process or methodology; (3) had inaccessible full text; or (4) were conference abstracts, dissertations, duplicate publications, or other non-original reports.

### 2.3 Study selection and screening

Study selection was conducted independently by two reviewers (An Liu and Nadeeka). After duplicate records had been removed, titles and abstracts were screened for eligibility. Full texts of potentially relevant studies were then assessed against the predefined inclusion and exclusion criteria. In addition, the reference lists of eligible articles were manually screened to identify any further relevant studies. Disagreements were resolved through discussion with a third reviewer until consensus was reached.

### 2.4 Data extraction

Data extraction was performed independently by two reviewers using a standardized form. Extracted information included general study characteristics, such as author, year of publication, study design, participant characteristics, data source, and sample size, as well as model-related information, including predictor selection, development algorithm, validation strategy, handling of missing data, processing of continuous variables, final predictors, and model performance. When multiple models were reported within a single study, the best-performing validated model, or the primary model specified by the authors, was selected for meta-analysis. When both development and validation performance estimates were available, validation estimates were prioritized.

### 2.5 Quality assessment

To assess the methodological quality and risk of bias in the included studies, we used the Predictive Modeling Risk of Bias Assessment Tool (PROBAST). Two investigators (An Liu and Nadeeka) independently evaluated the risk of bias and clinical applicability of each study using this validated framework, which is designed to rigorously appraise the development, validation, or refinement of individualized prediction models. The tool comprises 20 signaling questions categorized into four core domains: participants, predictors, outcomes, and analyses. Responses are classified as ‘yes,’ ‘probably yes,’ ‘no,’ ‘probably no,’ or ‘no information.’ A domain is designated as being at ‘high risk’ of bias if any single signaling question within that domain is answered with ‘no’ or ‘probably no,’ while an overall low risk of bias is assigned only when all domains meet the low-risk criteria.

### 2.6 Statistical analysis

To synthesize the performance of the validated models, we conducted a meta-analysis of the reported AUC values using R (version 4.4.2). Inter-study heterogeneity was rigorously evaluated utilizing the Cochrane Q test alongside the I^2^ index. Specifically, a random-effects model was employed to account for heterogeneity when I^2^ > 50%, whereas a fixed-effects model was deemed appropriate for instances where I^2^ < 50% (13). Furthermore, potential publication bias was scrutinized using Egger’s regression test, where a *p*-value > 0.05 suggested a minimal likelihood of significant publication bias influencing our synthesized results. When both development and validation performance were reported, only validation estimates were synthesized to reduce optimism bias.

## 3 Search results

### 3.1 Study selection

**Figure 1:**
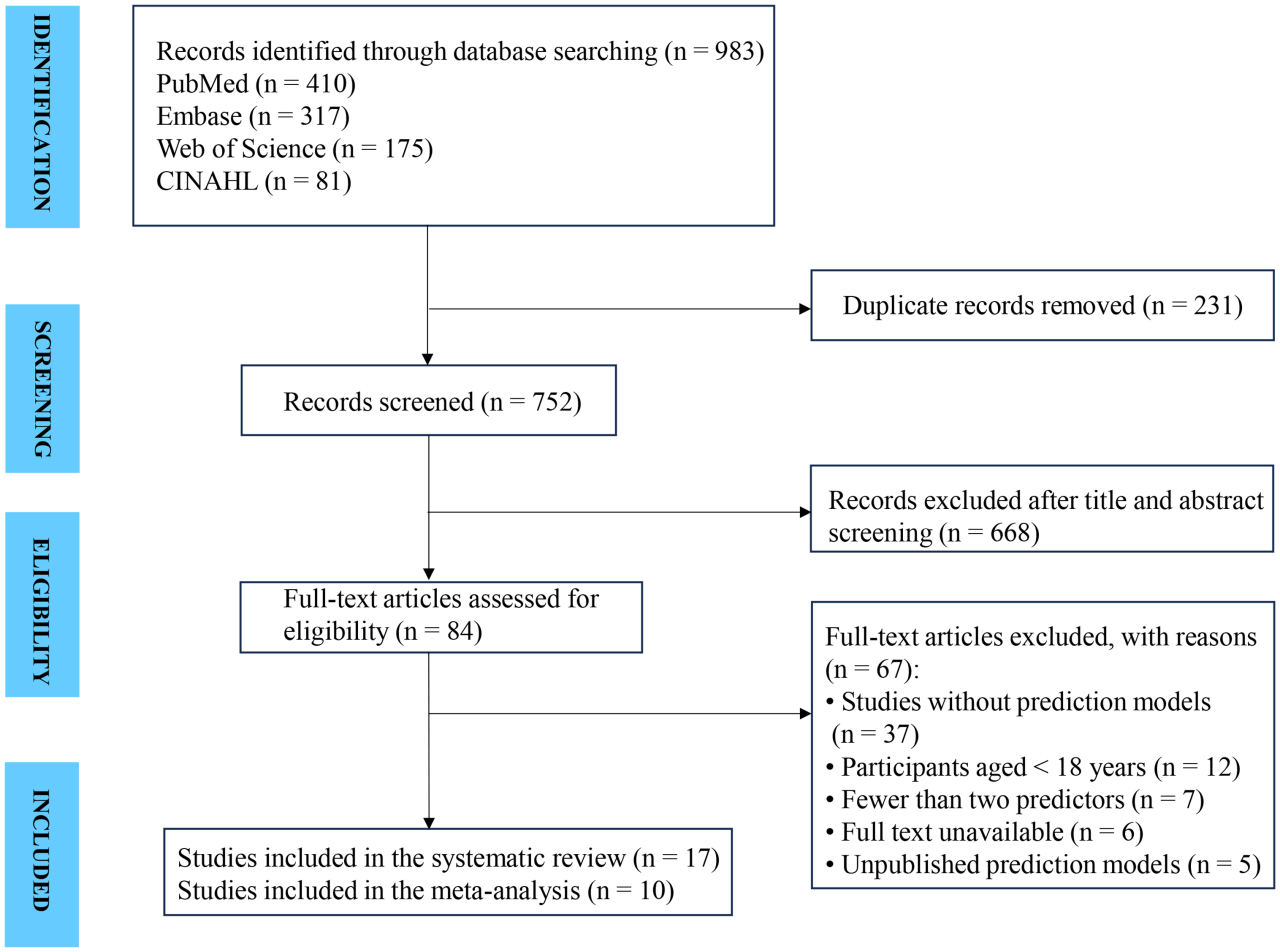
PRISMA flow diagram of the study selection process.

Figure 1 depicts the PRISMA flow chart that was employed in the selection process. A total of 983 papers were identified as potentially relevant through a literature search. Of these, 231 were duplicates and 688 were excluded after a review of the title and abstract. A further 67 were excluded after a review of the full text. The final number of articles included in this review was 17.

### 3.2 The general characteristics of the studies

Table 1 summarizes the study designs and participant characteristics of the 17 included studies. These studies were published between 2021 and 2025, with 14 conducted in China and three in the United States. Among the included studies, seven were cross-sectional studies, six were retrospective cohort studies, two were prospective cohort studies, and one study each was a mixed prospective-retrospective cohort study and a case–control study. Of the included studies, only four studies were multicenter and the rest were single-center. Regarding study populations, most studies included patients with a single disease, for example, gynecological cancers, lymphoma, hip fractures, stroke, radical gastrectomy, or thoracolumbar spine fractures. Three studies enrolled broader inpatient populations drawn from intensive care units, neurocritical care unit, or large hospital-based clinical databases. Sample sizes for all studies ranged from 419 to 94,642 individuals.

**Table 1:** Characteristics of included studies and outcomes.

Author (year)	Country	Research design	Participants	Data source	Main outcome
1. Wang et al. (20)	China	Cross-sectional study	673 patients undergoing surgery for gynecological cancer	Medical database	VTE/DVT
2. Ryan et al. (16)	United States	Cross-sectional study	94,642(12h)90,576(24h) patients from academic medical center	Electronic Medical Record System	DVT
3. Li et al. (21)	China	Prospective cohort study	420 patients from neurological ICU	Computerized clinical information system	DVT
4. Jin et al. (25)	China	Retrospective cohort study	1,035 patients with cancer	Electronic Medical Record System	DVT
5. Sheng et al. (37)	China	Cross-sectional study	3,078 patients from total hospital admissions	Medical database	VTE/DVT
6. Pan et al. (18)	China	Prospective cohort study	419 elderly patients with hip fracture	Medical database	DVT
7. Liu et al. (38)	China	Retrospective cohort study	429 patients with colorectal cancer	Medical database	DVT
8. Ma et al. (15)	United States	Cross-sectional study	13,025 patients with lymphoma	National Veterans Affairs healthcare system, Harris Health System, MD Anderson Cancer Center	VTE/DVT
9. Liu et al. (38)	China	Cross-sectional study	620 patients with stroke	Medical database	DVT
10. Shi et al. (24)	China	Mixed retrospective–prospective cohort study	809 patients with traumatic pelvic fracture	Medical database	DVT
11. Wei et al. (17)	China	Cross-sectional study	4424 patients with traumatic pelvic fracture	Medical database	DVT
12. Zhou et al. (19)	China	Retrospective case–control study	693 patients with gastric cancer who underwent radical gastrectomy	Medical database	DVT
13. Ma et al. (22)	China	Prospective cohort study	1350 patients with thoracolumbar fractures	Database of Surgical Site Infection in Orthopedic Surgery	DVT
14. Jin et al. (26)	China	Cross-sectional study	1494 patients from ICU	Electronic Medical Record System	VTE/DVT
15. Yang et al. (35)	China	Retrospective cohort study	568 patients with lumbar degenerative diseases	Medical database	DVT
16. Liu et al. (23)	China	Retrospective cohort study	3,116 patients with thoracic trauma (training cohort) 408 patients with thoracic trauma (external validation cohort)	Chest trauma cloud database.	DVT
17. Tabari et al. (14)	United States	Retrospective cohort study	21,549 patients with endovenous thermal ablation	National registry	DVT

As shown in Tables 2, 3, XGBoost, random forest, and logistic regression were the most frequently used modeling approaches among the included prediction models. Tree-based ensemble methods, particularly XGBoost and random forest, reported relatively high AUC values in several individual studies. However, the included studies differed in clinical populations, predictor sets, outcome definitions, and validation strategies, which should be considered when summarizing model performance. D-dimer, surgery, and age were the most commonly identified predictors across the included studies. The area under the curve (AUC) values for these models in the validation set ranged from 0.711 to 0.97. Fourteen models reported calibration results, with calibration plots being the most commonly used method.

**Table 2:** Methodological characteristics of included deep vein thrombosis prediction models.

Author (year)	DVT cases/sample size (%)	Missing data	Continuous variable processing	Variable selection
1. Wang et al. (20)	90/673 (13%)	Not reported	Not reported	Univariate logistic regression and multivariate logistic regression
2. Ryan et al. (16)	1230/94642 (1%) 999/90576 (1%)	Not reported	Median (IQR)	Not reported
3. Li et al. (21)	153/420 (36.4%)	Not reported	Median (IQR)	Univariate logistic regression and multivariate logistic regression
4. Jin et al. (25)	231/1035 (22.3%)	Median imputation	Median (IQR)	Univariate logistic regression/LASSO
5. Sheng et al. (37)	353/3078 (11.4%)	No missing data	Median (IQR)	Backwards elimination (RF XGBoost) and LASSO
6. Pan et al. (18)	128/419 (30.5%)	Not reported	Not reported	Univariate logistic regression and multivariate logistic regression
7. Liu et al. (38)	162/429 (37.77%)	KNN	Mean ± SD / Median (IQR)	Univariate regression analysis and RF/XGBoost/LASSO
8. Ma et al. (15)	13,025 (5.8–8.8%)	RF	Not reported	LASSO
9. Liu et al. (38)	A: 417/620 (67.3%) B: 203/620 (32.7)	RF	Mean ± SD / Median (IQR)	LASSO
10. Shi et al. (24)	216/809 (26.7%)	Not reported	Not reported	Univariate logistic regression/LASSO/Multivariate logistic regression
11. Wei et al. (17)	207/4424 (4.68%)	Multiple imputation	Median (IQR)	XGBoost
12. Zhou et al. (19)	38/693 (5.48%)	Not reported	Median (IQR)	Univariate logistic regression/Boruta algorithm/multivariate logistic regression
13. Ma et al. (22)	91/1350 (6%)	Not reported	Mean ± SD / Median (IQR)	Univariate analysis and multivariate logistic regression
14. Jin et al. (26)	389/1494 (26.04%)	Multiple imputation	Median (IQR)	Boruta algorithm
15. Yang et al. (35)	39/568 (6.87%)	Not reported	Not reported	LASSO and multivariate logistic regression
16. Liu et al. (23)	227/3524(6.4%)	Not reported	Not reported	BorutaShap algorithm
17. Tabari et al. (14)	345/21549 (1.59%)	Optimal Imputation	Mean ± SD	Not reported

**Table 3:** Performance analysis of deep vein thrombosis prediction models.

Author (year)	Model development method	Calibration method	Validation method	Final predictors	Model performance
1. Wang et al. (20)	LR	Calibration plot	Internal validation	Age/BMI/D-dimer /Surgery	A: AUC 0.803 B1: C-statistic 0.691
2. Ryan et al. (16)	SVM/XGBoost	Not reported	Not reported	Cancer status/VTE history/International normalized ratio (INR)/Change in INR and heart rate	A: XGBoost AUC 0.827(12 h) A: XGBoost AUC 0.851(24 h)
3. Li et al. (21)	LR	Brier score Calibration plots	Internal validation	Age/GCS score/D-dimer /Muscle strength and infection	A: LR AUC 0.817 B1: LR AUC 0.778
4. Jin et al. (25)	LDA/LR/CT/RF/SVM	Brier score Calibration plots	Internal validation	D-Dimer/Age/Charlson Comorbidity Index (CCI) Length of stay (LOS) and previously VTE history	B1: LDA AUC 0.773
5. Sheng et al. (37)	LR/RF/XGBoost	Hosmer-Lemeshow	Internal validation	Age/Central Venous catheter/Visible varicose veins and stroke	A: RF AUC 0.812 B1: RF AUC 0.804
6. Pan et al. (18)	LR	Hosmer-Lemeshow Calibration plot	Internal validation	Sex/Time from injury to admission/ASA classification/CRP elevation and D-dimer	A: AUC 0.750
7. Liu et al. (38)	LR/NB/GP/RF/XGBoost/MLP	Brier score, Calibration curves	Internal validation	Age/Preoperative albumin/ Preoperative white blood cell count/Surgery duration	A: XGBoost AUC 0.996 B1: XGBoost AUC 0.936
8. Ma et al. (15)	Fine-Gray competing risk models	Calibration plot	Internal validation External validation	Lymphoma subtype risk group/Treatment regimen/BMI ≥ 35/Recent hospitalization/History of VTE	A: C-statistic 0.68 B1: C-statistic 0.69 B2: C-statistic 0.72
9. Liu et al. (38)	LR/RF/XGBoost/SVM/NN/DT/NB/KNN	Hosmer-Lemeshow	Internal validation	D-dimer/Age/Brunnstrom stage /Prothrombin time and mobility ability	A: RF AUC 0.74 B1: RF AUC 0.73
10. Shi et al. (24)	DRNS	Hosmer-Lemeshow Calibration plot	Internal validation	Age≥40/Combined femoral fractures/BMI/ISS score at admission	A: DRNS AUC 0.748 B1: DRNS AUC 0.920
11. Wei et al. (17)	LR/RF/XGBoost/SVM/MLP	Not reported	Not reported	Age/Hypertension/ Fibrinogen/Surgical grade/Platelets	A: XGBoost AUC 0.979
12. Zhou et al. (19)	LR/DT/RF/SVM/XGBoost/ LightGBM	Calibration plots	Internal validation	Age/D-dimer/LDL/CA125/ CA^2+^/Cl^−^	A: LR AUC 0.936 B1: LR AUC 0.875
13. Ma et al. (22)	LR	Hosmer-Lemeshow	Internal validation	Surgery/Number of fractured vertebrae/Chest injury/Lower extremity fractures/ASIA (Grade A/B)	A: LR AUC 0.876 B1: LR AUC 0.853
14. Jin et al. (26)	LR/RF/XGBoost/SVM/ GBDT	Hosmer-Lemeshow	Internal validation	Age/Length of braking time/ D-dimer/Activated partial thromboplastin time/ Mechanical ventilation time	A: RF AUC 1 B1: RF AUC 0.788
15. Yang et al. (35)	LR	Calibration plots	Internal validation	Age/Walking impairment /Diabetes mellitus/ Activated partial thromboplastin time (APTT) and D-dimer	A: LR AUC 0.87 B1: LR AUC 0.97
16. Liu et al. (23)	LR/RF/SVM/MLP/GBM	Calibration plots	Internal validation External validation	Age/BMI/Number of broken ends of rib fracture/ Rib fracture surgery/Multiple traumas/Lower limb fracture/ Tracheal Intubation/ Blood transfusion/D-D 24 h/ PT 24 h/Plt 24 h and Hb 24 h.	A: RF AUC 0.997 B1: RF AUC 0.83 B2: RF AUC 0.879
17. Tabari et al. (14)	CART/RF/OCT/ XGBoost	Not reported	Internal validation	No feature selection; all 34 variables included.	A: XGBoost AUC 1 B1: XGBoost AUC 0.711

A, model development cohort (excluded from meta-analysis); B1, internal validation cohort; B2, external validation cohort; KNN, k-nearest neighbor; MLP, Multilayer Perceptron; GBDT, Gradient Boosting Decision Tree; DRNS, Deep Residual Neural Networks; NN, Neural Networks; DT, Decision Tree; NB, Naive Bayes; CART, Classification and Regression Trees; OCT, Optimal Classification Trees; AUC, area under the receiver operating characteristic curve, Performance interpretation: AUC 0.5–0.7, poor; 0.7–0.8, moderate; 0.8–0.9, good; 0.9–1.0, excellent.

### 3.3 Model validation

Among the included studies, 14 performed internal validation. The models developed by Ma and Tabari underwent both internal and external validation (14, 15), whereas the models reported by Wei and Ryan did not report either internal or external validation (16, 17). Pan reported conducting internal validation but did not provide the corresponding AUC value (18).

### 3.4 Results of quality assessment

Table 4 summarize the risk of bias and applicability concerns across the included studies. Six studies were judged to be at high risk of bias, and six were rated as having an unclear risk of bias due to insufficient reporting, indicating that methodological limitations were common during model development or validation.

**Table 4:** PROBAST results of the included studies.

Author (year)	Study type	ROB				Applicability			Overall	
		Participants	Predictors	Outcome	Analysis	Participants	Predictors	Outcome	ROB	Applicability
Wang et al. (20)	B	+	+	+	?	+	+	+	?	+
Ryan et al. (16)	B	+	+	+	_	+	+	+	_	+
Li et al. (21)	B	+	+	+	?	+	+	+	?	+
Jin et al. (25)	B	+	+	+	+	+	_	+	+	_
Sheng et al. (37)	B	+	+	+	+	+	+	+	+	+
Pan et al. (18)	B	+	+	+	_	+	+	+	_	+
Liu et al. (38)	B	+	+	+	+	+	+	+	+	+
Ma et al. (15)	B	+	+	+	?	+	+	+	?	+
Liu et al. (38)	B	+	+	+	+	+	+	+	+	+
Shi et al. (24)	B	+	+	+	?	+	_	+	?	_
Wei et al. (17)	A	_	+	+	_	+	+	+	_	+
Zhou et al. (19)	B	_	+	_	_	+	_	+	_	_
Ma et al. (22)	B	+	+	+	?	+	_	+	?	_
Jin et al. (26)	B	+	+	+	+	+	_	+	+	_
Yang et al. (35)	B	+	+	+	_	+	+	_	_	+
Liu et al. (23)	B	+	+	+	?	+	+	+	?	+
Tabari et al. (14)	B	+	_	_	_	+	+	+	_	+

PROBAST, Prediction model Risk of Bias Assessment Tool; ROB, risk of bias. A indicates “development only”; B indicates “development and validation in the same publication”. + indicates low ROB/low concern regarding applicability; − indicates high ROB/high concern regarding application; ? indicates unclear ROB/unclear concern regarding applicability (Supplementary Table S1).

In the participant domain, two studies were identified as being at high risk of bias, mainly due to the use of inappropriate data sources (17, 19). In the predictor domain, one study was judged to have a high risk of bias because it did not perform feature selection. The predictors were not predefined but rather selected in a data-driven manner during model development, which may have led to overfitting or model dependence on sample-specific characteristics, thereby limiting generalizability (14). In the outcome domain, two studies were judged to have a high risk of bias. One study did not exclude predictor variables from the outcome definition, and the outcome assessment was closely related to the predictors (19). The other study failed to describe the process of predictor selection or inclusion; the absence of predefined variable selection criteria may have introduced data-driven bias and overfitting, thereby reducing the reliability of predictive performance and limiting the external generalizability of the model (14).

In the analysis domain, six studies were assessed as having a high risk of bias, with six studies providing incomplete or unclear descriptions of the analytical procedures involved. Among these studies, one had an inadequate sample size, failing to meet the recommended guideline of more than 10 events per variable (EPV) (19). The study by Tabari did not include any feature selection procedure (14). One study demonstrated inappropriate handling of data exclusion and failed to explain the occurrence or management of missing data (18). Additionally, three studies did not consider or report model calibration methods (14, 16, 17), which may compromise the robustness of the predictive models. In terms of unclear reporting, six studies did not explain the handling of missing data or the procedures used to process categorical or continuous variables (15, 20–23).

Regarding applicability, 12 studies were rated as having low concern, while five were judged to have high applicability concerns. In the participants domain, all studies focused on hospitalized patients, showing high consistency with our review’s target population. However, high applicability concerns were identified in the predictors and outcome domains. Specifically, in predictors domain, four studies were assessed as having high concerns: two due to the non-standardized timing of predictor measurements—which failed to align with the intended clinical decision window—and two because the required variables involved specialized diagnostic tests that are not readily available in routine clinical practice (19, 22, 24, 25). In the outcome domain, one study was deemed high-risk due to the aggregation of disparate clinical events (i.e., deep vein thrombosis and pulmonary embolism) into a single composite outcome, which limits the model’s utility for predicting specific venous thromboembolism complications (26).

### 3.5 Meta-analysis

**Figure 2:**
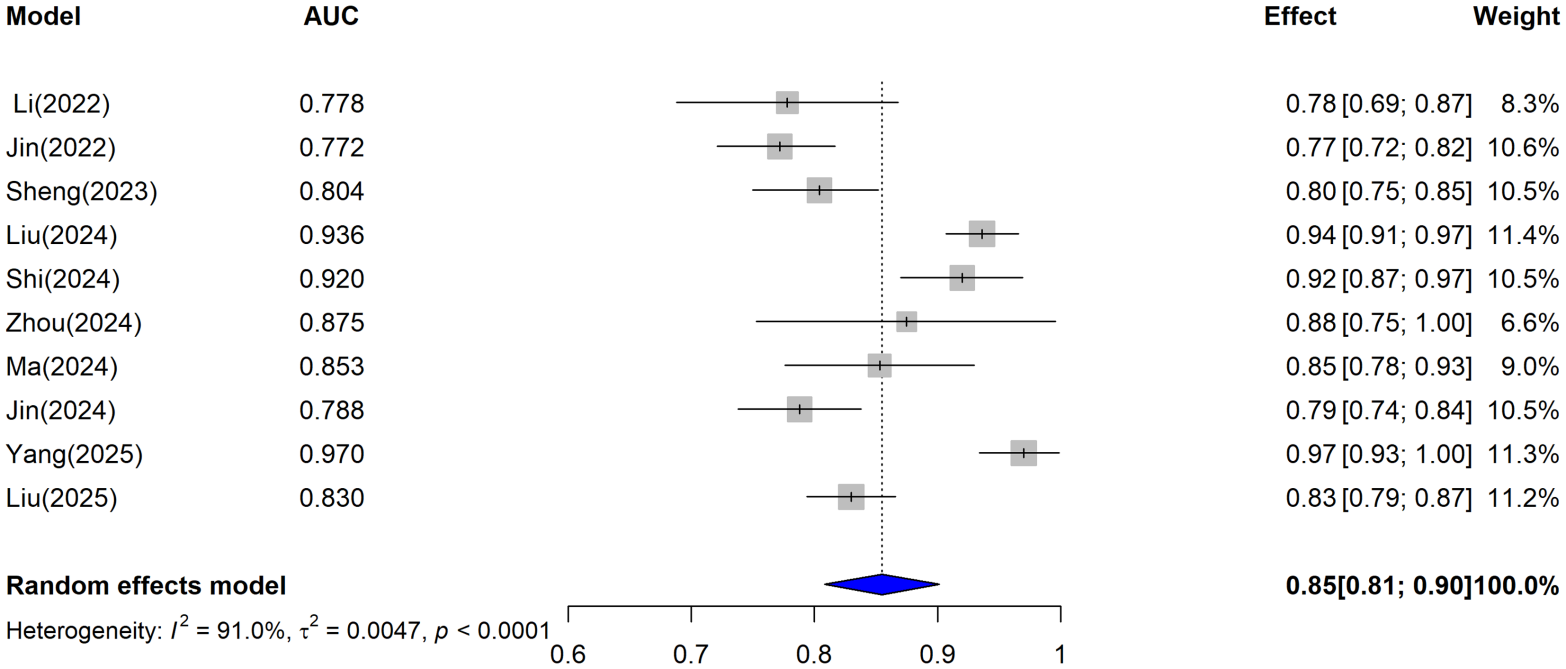
Forest plot of the random effects meta-analysis of the combined AUC estimates for the 10 validated models.

Due to inadequate reporting of model development details among the candidate studies, only 10 studies met the inclusion criteria for this meta-analysis. Specifically, the studies by Li (2021) and Ma (2024) were excluded because they did not report validation AUC estimates suitable for quantitative synthesis. Furthermore, the studies by Ryan (2021), Pan (2023), and Wei (2024) failed to report AUC values for either internal or external validation. Lastly, Liu (2024) and Tabari (2025) were excluded due to the absence of reported 95% confidence intervals (CIs) for their AUC estimates. A random-effects model was employed to calculate the pooled AUC, yielding a value of 0.85 (95% CI: 0.81–0.90; Figure 2). Although substantial heterogeneity was observed (I^2^ = 91%, *p* < 0.05), the 95% prediction interval ranged from 0.81 to 0.90, suggesting acceptable average discrimination across included validation datasets; however, interpretation remains limited by substantial unexplained heterogeneity. Additionally, Egger’s test showed no significant publication bias (intercept = −0.919, *p* = 0.358).

Even after subgrouping by modeling algorithm, high levels of heterogeneity persisted in both the logistic regression (LR) group (I^2^ = 92.5%) and the other model group (I^2^ = 91%). The test for subgroup differences was not significant (*p* = 0.92). These findings indicate that the choice of modeling algorithm was not the primary driver of the observed heterogeneity (Supplementary Figure S1).

Sensitivity analysis indicated that no single study disproportionately influenced the overall heterogeneity, as the I^2^ value remained consistently high across all leave-one-out iterations (Supplementary Table S2).

## 4 Discussion

Deep vein thrombosis (DVT) remains an important complication among hospitalized patients, and early risk identification is essential for timely prevention and management (27). In this review, we found that machine learning–based prediction models for DVT have increased in recent years, particularly in Chinese cohorts, and generally show promising discriminatory performance. However, important methodological limitations remain in both model development and validation (28, 29).

We identified 17 studies on DVT prediction models across diverse clinical settings, of which 10 were included in the meta-analysis. The pooled AUC was 0.85 (95% CI: 0.81–0.90), suggesting relatively favorable discriminatory performance among the models included in the meta-analysis. However, model quality remains a major concern. Based on PROBAST, six studies were judged to have a high risk of bias, while five showed limited applicability to broader clinical contexts.

This variability was also reflected in the meta-analysis, which showed substantial heterogeneity (I^2^ = 91%). To explore potential sources of this heterogeneity, we performed subgroup and sensitivity analyses. Subgroup stratification by modeling algorithms (LR vs. other approaches) did not reduce heterogeneity, and leave-one-out sensitivity analysis indicated that the pooled estimates were not driven by any single study. These findings indicate that the observed heterogeneity was more likely related to broader cross-study differences than to modeling algorithm alone.

Several factors may explain this heterogeneity. First, variations in patient case-mix across the included studies represent a primary source of clinical heterogeneity. For instance, patients with thoracic trauma typically present with acute traumatic risk factors and sudden immobility (23), whereas those with colorectal cancer exhibit a more insidious, chronic hypercoagulable state driven by malignant systemic inflammation (1). Such fundamental disparities in baseline risk profiles among diverse clinical departments inherently influence the models’ discriminative power (AUC). Second, inconsistencies in predictor definitions and temporal windows further exacerbate the observed variance. Specifically, Shi et al. restricted their cohort to patients over 40 years of age with a trauma-to-admission interval of < 48 h (24), whereas Ma et al. utilized data collected strictly within 24 h of admission (22). These divergent temporal windows and inclusion criteria introduce significant methodological noise, thereby contributing to the heterogeneity in predictive performance. However, such heterogeneity limits the interpretability of the pooled results and warrants cautious interpretation (30, 31).

Regarding model performance, ensemble learning methods, particularly XGBoost, showed favorable discrimination in several inpatient settings. This may be related to their ability to handle high-dimensional clinical data and capture nonlinear relationships among predictors derived from electronic medical records (32, 33). However, strong discrimination alone does not guarantee clinical usefulness. Calibration, external validation, interpretability, and implementation feasibility are equally important for routine adoption. In addition, the apparent advantage of complex models should be interpreted cautiously, as some high AUC values were reported in relatively small or single-center datasets, where overfitting remains a concern (34). From a clinical perspective, prediction models for DVT may support early risk stratification and individualized preventive decision-making in hospitalized populations. Nevertheless, their current clinical applicability remains limited by insufficient external validation, incomplete reporting of calibration and clinical utility, and unclear implementation pathways (35). These issues should be addressed before such models can be reliably translated into routine clinical practice.

Another important barrier to clinical adoption is model interpretability. Although complex machine learning algorithms may improve predictive performance in complex clinical settings, their “black-box” nature can limit clinician acceptance (36). While interpretability tools such as SHAP or LIME can help explain model outputs, only six studies in our review reported using such approaches (1, 14, 16, 26, 37, 38). This suggests that interpretability remains under-addressed in the current literature and should receive greater attention in future model development.

Furthermore, our review identified D-dimer, surgery, and age as the most frequently retained predictors across models. This is clinically plausible. D-dimer reflects activation of coagulation and fibrinolysis and remains one of the most widely used biomarkers in thrombosis assessment (39). Surgery is a well-established trigger for venous thromboembolism because it contributes to immobility, tissue injury, and inflammatory activation, all of which promote thrombogenesis (40). Age is also consistently associated with increased thrombotic risk, likely reflecting the combined effects of reduced mobility, comorbidity burden, and age-related vascular vulnerability (41). The repeated inclusion of these variables across different models suggests that, despite heterogeneity in patient population and algorithm choice, several core predictors remain central to DVT risk stratification.

In conclusion, machine learning–based prediction models for DVT in hospitalized patients show encouraging discriminatory performance, but the current evidence remains constrained by heterogeneity, risk of bias, incomplete reporting, and limited external validation. At present, these models should be regarded as promising rather than practice-ready. Future research should prioritize transparent reporting, stronger validation, and clinically meaningful comparison with existing risk assessment approaches.

### 4.1 Limitations

This review has several limitations. First, substantial between-study heterogeneity limited the interpretability and comparability of the pooled estimates. Second, the meta-analysis was based on a limited number of studies with sufficient validation data, which may reduce the precision and robustness of the summary estimates. Third, the geographic concentration of the included studies (mainly mainland China) may limit the applicability of our findings to other healthcare settings with different patient demographics or clinical practices. Fourth, restricting our search to English-language publications may have introduced language bias. Finally, inconsistent reporting across the primary studies precluded a more comprehensive assessment of calibration, clinical utility, and transportability.

## 5 Conclusion

This systematic review and meta-analysis of 17 studies, including 10 with sufficient validation data, showed that machine learning-based approaches had good discriminatory performance for DVT, with a pooled AUC of 0.85 (95% CI: 0.81–0.90). Despite this promise, the field is characterized by substantial methodological heterogeneity (I^2^ = 91%) with risk of bias most commonly arising from limitations in the analysis domain. Our findings identified D-dimer, surgery, and age as the most consistently retained predictors across studies. To facilitate clinical adoption, future research should shift focus from redundant model development to prospective, multi-center external validation and head-to-head comparisons with established clinical scores. Adherence to TRIPOD+AI and PROBAST+AI reporting standards will be essential to ensuring that these high-dimensional models provide reliable, transparent, and generalizable support for venous thromboembolism prevention in the digital health era.

## Data Availability

The original contributions presented in the study are included in the article/Supplementary material, further inquiries can be directed to the corresponding author.
