# Supplementary material for: Machine learning prediction models for deep vein thrombosis in hospitalized patients: a systematic review and meta-analysis
Source: Front Med (Lausanne). 2026 May 26;13:1849096. doi: 10.3389/fmed.2026.1849096 (PMC13246386; doi:10.3389/fmed.2026.1849096)
Supplement: Supplementary file 1 [file Supplementary_file_1.DOCX]

Supplementary Material S1: Detailed Search Strategies for All Databases

Search Strategy Overview A comprehensive and systematic literature search was performed across four international databases: PubMed, Embase, Web of Science, and the Cumulative Index to Nursing and Allied Health Literature (CINAHL). The search spanned from November 1, 2015 to November 1, 2025, and was restricted to English-language publications. The search strategy utilized a combination of controlled vocabulary (MeSH/Emtree) and free-text keywords. Boolean operators (AND, OR) were applied to integrate terms related to (1) machine learning and prediction modeling, and (2) deep vein thrombosis (DVT).

1. PubMed Search Date: November 1, 2025, Search String: (("Machine Learning"[MeSH Terms] OR "Artificial Intelligence"[MeSH Terms] OR "machine learning"[Title/Abstract] OR "ML"[Title/Abstract] OR "predictive modeling"[Title/Abstract] OR "predictive modelling"[Title/Abstract] OR "clinical prediction models"[Title/Abstract] OR "algorithm*"[Title/Abstract]))

AND (("Venous Thrombosis"[MeSH Terms] OR "Deep Vein Thrombosis"[Title/Abstract] OR "DVT"[Title/Abstract] OR "phlebothrombosis"[Title/Abstract] OR "lower extremity deep vein thrombosis"[Title/Abstract] OR "lower extremity deep venous thrombosis"[Title/Abstract]))

AND (English[Language])

2. Embase Search Date: November 1, 2025, Search String: ('machine learning'/exp OR 'artificial intelligence'/exp OR 'machine learning':ti,ab OR 'ml':ti,ab OR 'predictive modeling':ti,ab OR 'predictive modelling':ti,ab OR 'clinical prediction model':ti,ab OR 'algorithm*':ti,ab)

AND ('deep vein thrombosis'/exp OR 'phlebothrombosis'/exp OR 'deep vein thrombosis':ti,ab OR 'dvt':ti,ab OR 'lower extremity deep vein thrombosis':ti,ab)

AND ([English]/lim)

3. Web of Science (Core Collection) Search Date: November 1, 2025, Search String: TS= ("machine learning" OR "ML" OR "predictive modeling" OR "predictive modelling" OR "clinical prediction models" OR "algorithm*")

AND TS= ("deep vein thrombosis" OR "DVT" OR "phlebothrombosis" OR "lower extremity deep vein thrombosis")

AND LA= (English)

4. CINAHL (via EBSCO) Search Date: November 1, 2025, Search String: ((MH "Machine Learning+") OR (MH "Artificial Intelligence+") OR TI "machine learning" OR AB "machine learning" OR TI "ML" OR AB "ML" OR TI "predictive modeling" OR AB "predictive modeling" OR TI "clinical prediction models" OR AB "clinical prediction models")

AND ((MH "Venous Thrombosis+") OR TI "deep vein thrombosis" OR AB "deep vein thrombosis" OR TI "DVT" OR AB "DVT" OR TI "phlebothrombosis" OR AB "phlebothrombosis" OR TI "lower extremity deep vein thrombosis" OR AB "lower extremity deep vein thrombosis")

AND (Limiters: English Language)


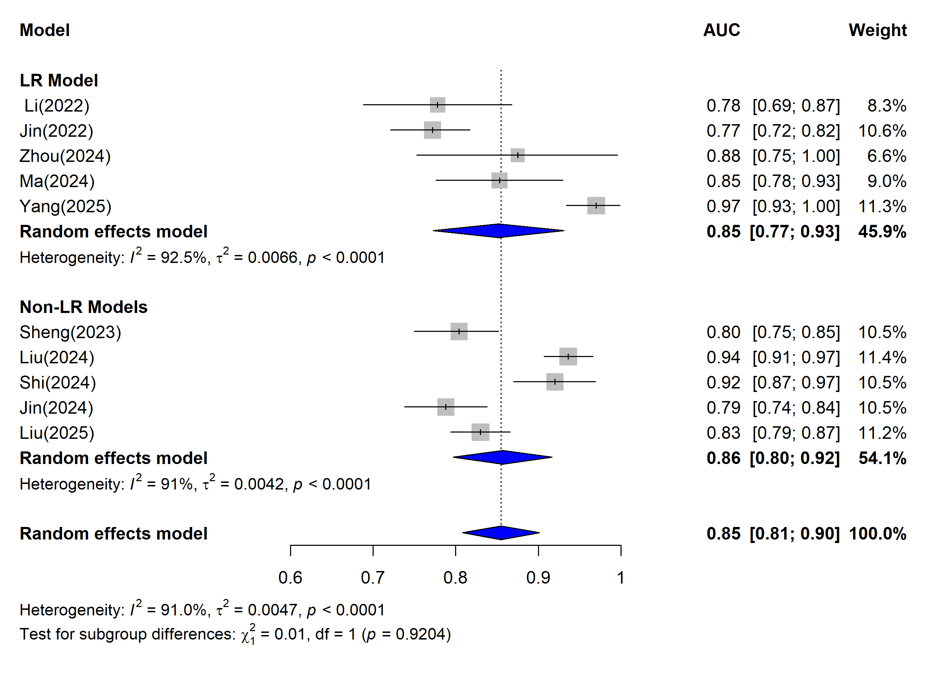


**Supplementary Figure S1.** **Subgroup analysis among different models**

| Study | Effect | Lower | Upper | I^2^ |
| --- | --- | --- | --- | --- |
| Li(2022) | 0.862 | 0.813 | 0.911 | 91.544 |
| Jin(2022) | 0.865 | 0.817 | 0.913 | 89.809 |
| Sheng(2023) | 0.861 | 0.810 | 0.911 | 91.160 |
| Liu(2024) | 0.845 | 0.796 | 0.893 | 90.131 |
| Shi(2024) | 0.847 | 0.797 | 0.897 | 91.734 |
| Zhou(2024) | 0.853 | 0.803 | 0.903 | 91.968 |
| Ma(2024) | 0.855 | 0.803 | 0.906 | 91.931 |
| Jin(2024) | 0.863 | 0.813 | 0.912 | 90.658 |
| Yang(2025) | 0.841 | 0.798 | 0.883 | 87.199 |
| Liu(2025) | 0.858 | 0.806 | 0.910 | 91.211 |

**Supplementary Table S2. Leave-one-out sensitivity analysis of pooled AUC and heterogeneity**
