# Supplementary material for: Machine learning prediction models for deep vein thrombosis in hospitalized patients: a systematic review and meta-analysis
Source: Front Med (Lausanne). 2026 May 26;13:1849096. doi: 10.3389/fmed.2026.1849096 (PMC13246386; doi:10.3389/fmed.2026.1849096)
Supplement: Supplementary file 2 [file Table_1.docx]

**Supplementary Table S1: Prediction Model Risk of Bias Assessment Tool**

| Author (year) | Study type |  | 1.1 Were appropriate data sources used, e.g. cohort, RCT or nested case-control study data? | |  |  |  | 1.2 Were all inclusions and exclusions of participants appropriate? | |  |
| --- | --- | --- | --- | --- | --- | --- | --- | --- | --- | --- |
|  |  |  | 1.1 | Notes 1.1 | |  |  | 1.2 | Notes 1.2 | |
| Li Wang  (2021) | Development only |  | Yes | Cross-sectional study | |  |  | Yes | Suitable samples | |
| Logan Ryan  (2021) | Development only |  | Yes | Cross-sectional study | |  |  | Yes | Suitable samples | |
| Rongqing Li (2022) | Development only |  | Yes | Prospective cohort study | |  |  | Yes | Suitable samples | |
| Shuai Jin  (2022) | Development only |  | Yes | Retrospective cohort study | |  |  | Yes | Suitable samples | |
| Wenbo Sheng  (2023) | Development only |  | Yes | Cross-sectional study | |  |  | Yes | Suitable samples | |
| Sheng Pan  (2023) | Development only |  | Yes | Prospective cohort study | |  |  | Yes | Suitable samples | |
| Xin Liu  (2024) | Development only |  | Yes | Retrospective cohort study | |  |  | Yes | Suitable samples | |

**Supplementary Table S1: Continued**

| Author (year) | Study type |  | 1.1 Were appropriate data sources used, e.g. cohort, RCT or nested case-control study data? | |  |  |  | 1.2 Were all inclusions and exclusions of participants appropriate? | |  | |
| --- | --- | --- | --- | --- | --- | --- | --- | --- | --- | --- | --- |
|  |  |  | 1.1 | Notes 1.1 | |  |  | 1.2 | Notes 1.2 | |  |
| Shengling Ma  (2024) | Development with external validation |  | Yes | Cross-sectional study | |  |  | Yes | Suitable samples | |  |
| Lingling Liu  (2024) | Development only |  | Yes | Cross-sectional study | |  |  | Yes | Suitable samples | |  |
| Dongcheng Shi  (2024) | Development only |  | Yes | Retrospective and prospective | |  |  | Yes | Suitable samples | |  |
| Conghui Wei  (2024) | Development only |  | Yes | Cross-sectional study | |  |  | No | Absence of exclusion criteria | |  |
| Haiyan Zhou  (2024) | Development only |  | No | Case contral | |  |  | Yes | Suitable samples | |  |
| Jiangtao Ma  (2024) | Development only |  | Yes | Prospective cohort study | |  |  | Yes | Suitable samples | |  |
| Jie Jin  (2024) | Development only |  | Yes | Cross-sectional study | |  |  | Yes | Suitable samples | |  |

**Supplementary Table S1: Continued**

| Author (year) | Study type |  | 1.1 Were appropriate data sources used, e.g. cohort, RCT or nested case-control study data? | |  |  |  | 1.2 Were all inclusions and exclusions of participants appropriate? | |  | |
| --- | --- | --- | --- | --- | --- | --- | --- | --- | --- | --- | --- |
|  |  |  | 1.1 | Notes 1.1 | |  |  | 1.2 | Notes 1.2 | |  |
| Tao Yang  (2025) | Development only |  | Yes | Retrospective cohort study | |  |  | Yes | Suitable samples | |  |
| Kaibin Liu  (2025) | Development only |  | Yes | Retrospective cohort study | |  |  | Yes | Suitable samples | |  |
| Azadeh Tabari  (2025) | Development with external validation |  | Yes | Retrospective cohort study | |  |  | Yes | Suitable samples | |  |

**Supplementary Table S1: Continued**

| Author (year) | Study type |  | Domain-level  RoB judgement | |  |  |  | Domain-level applicability judgement | |  |
| --- | --- | --- | --- | --- | --- | --- | --- | --- | --- | --- |
|  |  |  | Reviewer's Judgement | RoB note | |  |  | Applicability | App note | |
| Li Wang  (2021) | Development only |  | Low | Cross-sectional study | |  |  | Low concerns | The study population consists of patients during hospitalization, which aligns with the review's objective. | |
| Logan Ryan  (2021) | Development only |  | Low | Cross-sectional study | |  |  | Low concerns | The study population consists of patients during hospitalization, which aligns with the review's objective. | |
| Rongqing Li (2022) | Development only |  | Low | Prospective cohort study | |  |  | Low concerns | The study population consists of patients during hospitalization, which aligns with the review's objective. | |
| Shuai Jin  (2022) | Development only |  | Low | Retrospective cohort study | |  |  | Low concerns | The study population consists of patients during hospitalization, which aligns with the review's objective. | |
| Wenbo Sheng  (2023) | Development only |  | Low | Cross-sectional study | |  |  | Low concerns | The study population consists of patients during hospitalization, which aligns with the review's objective. | |
| Sheng Pan  (2023) | Development only |  | Low | Prospective cohort study | |  |  | Low concerns | The study population consists of patients during hospitalization, which aligns with the review's objective. | |
| Xin Liu  (2024) | Development only |  | Low | Retrospective cohort study | |  |  | Low concerns | The study population consists of patients during hospitalization, which aligns with the review's objective. | |

**Supplementary Table S1: Continued**

| Author (year) | Study type |  | Domain-level  RoB judgement | |  |  |  | Domain-level applicability judgement | |  | |
| --- | --- | --- | --- | --- | --- | --- | --- | --- | --- | --- | --- |
|  |  |  | Reviewer's Judgement | RoB note | |  |  | Applicability | App note | |  |
| Shengling Ma  (2024) | Development with external validation |  | Low | Cross-sectional study | |  |  | Low concerns | The study population consists of patients during hospitalization, which aligns with the review's objective. | |  |
| Lingling Liu  (2024) | Development only |  | Low | Cross-sectional study | |  |  | Low concerns | The study population consists of patients during hospitalization, which aligns with the review's objective. | |  |
| Dongcheng Shi  (2024) | Development only |  | Low | Retrospective and prospective | |  |  | Low concerns | The study population consists of patients during hospitalization, which aligns with the review's objective. | |  |
| Conghui Wei  (2024) | Development only |  | High | Absence of exclusion criteria | |  |  | Low concerns | The study population consists of patients during hospitalization, which aligns with the review's objective. | |  |
| Haiyan Zhou  (2024) | Development only |  | High | case contral | |  |  | Low concerns | The study population consists of patients during hospitalization, which aligns with the review's objective. | |  |
| Jiangtao Ma  (2024) | Development only |  | Low | Prospective cohort study | |  |  | Low concerns | The study population consists of patients during hospitalization, which aligns with the review's objective. | |  |
| Jie Jin  (2024) | Development only |  | Low | Cross-sectional study | |  |  | Low concerns | The study population consists of patients during hospitalization, which aligns with the review's objective. | |  |

**Supplementary Table S1: Continued**

| Author (year) | Study type |  | Domain-level  RoB judgement | |  |  |  | Domain-level applicability judgement | |  | |
| --- | --- | --- | --- | --- | --- | --- | --- | --- | --- | --- | --- |
|  |  |  | Reviewer's Judgement | RoB note | |  |  | Applicability | App note | |  |
| Tao Yang  (2025) | Development only |  | Low | Retrospective cohort study | |  |  | Low concerns | The study population consists of patients during hospitalization, which aligns with the review's objective. | |  |
| Kaibin Liu  (2025) | Development only |  | Low | Retrospective cohort study | |  |  | Low concerns | The study population consists of patients during hospitalization, which aligns with the review's objective. | |  |
| Azadeh Tabari  (2025) | Development with external validation |  | Low | Retrospective cohort study | |  |  | Low concerns | The study population consists of patients during hospitalization, which aligns with the review's objective. | |  |

**Supplementary Table S1: Continued**

| Author (year) | Study type |  | 2.1 Were predictors defined and assessed in a similar way for all participants? | |  |  |  | 2.2 Were predictor assessment made without knowledge of outcome data? | |  |
| --- | --- | --- | --- | --- | --- | --- | --- | --- | --- | --- |
|  |  |  | 2.1 | Notes 2.1 | |  |  | 2.2 | Notes 2.2 | |
| Li Wang  (2021) | Development only |  | Yes | All measurement units shall adopt the same standard. | |  |  | Yes | Objectively recorded variables | |
| Logan Ryan  (2021) | Development only |  | Yes | All measurement units shall adopt the same standard. | |  |  | Yes | Objectively recorded variables | |
| Rongqing Li (2022) | Development only |  | Yes | All measurement units shall adopt the same standard. | |  |  | Yes | Objectively recorded variables | |
| Shuai Jin  (2022) | Development only |  | Yes | All measurement units shall adopt the same standard. | |  |  | Yes | Objectively recorded variables | |
| Wenbo Sheng  (2023) | Development only |  | Yes | All measurement units shall adopt the same standard. | |  |  | Yes | Objectively recorded variables | |
| Sheng Pan  (2023) | Development only |  | Yes | All measurement units shall adopt the same standard. | |  |  | Yes | Objectively recorded variables | |
| Xin Liu  (2024) | Development only |  | Yes | All measurement units shall adopt the same standard. | |  |  | Yes | Objectively recorded variables | |

**Supplementary Table S1: Continued**

| Author (year) | Study type |  | 2.1 Were predictors defined and assessed in a similar way for all participants? | |  |  |  | 2.2 Were predictor assessment made without knowledge of outcome data? | |  | |
| --- | --- | --- | --- | --- | --- | --- | --- | --- | --- | --- | --- |
|  |  |  | 2.1 | Notes 2.1 | |  |  | 2.2 | Notes 2.2 | |  |
| Shengling Ma  (2024) | Development with external validation |  | Yes | All measurement units shall adopt the same standard. | |  |  | Yes | Objectively recorded variables | |  |
| Lingling Liu  (2024) | Development only |  | Yes | All measurement units shall adopt the same standard. | |  |  | Yes | Objectively recorded variables | |  |
| Dongcheng Shi  (2024) | Development only |  | Yes | All measurement units shall adopt the same standard. | |  |  | Yes | Objectively recorded variables | |  |
| Conghui Wei  (2024) | Development only |  | Yes | All measurement units shall adopt the same standard. | |  |  | Yes | Objectively recorded variables | |  |
| Haiyan Zhou  (2024) | Development only |  | Yes | All measurement units shall adopt the same standard. | |  |  | Yes | Objectively recorded variables | |  |
| Jiangtao Ma  (2024) | Development only |  | Yes | All measurement units shall adopt the same standard. | |  |  | Yes | Objectively recorded variables | |  |
| Jie Jin  (2024) | Development only |  | Yes | All measurement units shall adopt the same standard. | |  |  | Yes | Objectively recorded variables | |  |

**Supplementary Table S1: Continued**

| Author (year) | Study type |  | 2.1 Were predictors defined and assessed in a similar way for all participants? | |  |  |  | 2.2 Were predictor assessment made without knowledge of outcome data? | |  | |
| --- | --- | --- | --- | --- | --- | --- | --- | --- | --- | --- | --- |
|  |  |  | 2.1 | Notes 2.1 | |  |  | 2.2 | Notes 2.2 | |  |
| Tao Yang  (2025) | Development only |  | Yes | All measurement units shall adopt the same standard. | |  |  | Yes | Objectively recorded variables | |  |
| Kaibin Liu  (2025) | Development only |  | Yes | All measurement units shall adopt the same standard. | |  |  | Yes | Objectively recorded variables | |  |
| Azadeh Tabari  (2025) | Development with external validation |  | No | Predictors are not predefined but are selected in a data-driven manner during the modelling process. | |  |  | Yes | Objectively recorded variables | |  |

**Supplementary Table S1: Continued**

| Author (year) | Study type |  | 2.3 Are all predictors available at the time the model is intended to be used? | |  |  |  | Domain-level  RoB judgement | |  |
| --- | --- | --- | --- | --- | --- | --- | --- | --- | --- | --- |
|  |  |  | 2.3 | Notes 2.3 | |  |  | Reviewer's Judgement | RoB note | |
| Li Wang  (2021) | Development only |  | Yes | Available for use at any time | |  |  | Low | Predictors were consistently defined and measured before diagnosis using routine clinical data; no subjective reassessment was performed. | |
| Logan Ryan  (2021) | Development only |  | Yes | Available for use at any time | |  |  | Low | Predictors were consistently defined and measured before diagnosis using routine clinical data; no subjective reassessment was performed. | |
| Rongqing Li (2022) | Development only |  | Yes | Available for use at any time | |  |  | Low | Predictors were consistently defined and measured before diagnosis using routine clinical data; no subjective reassessment was performed. | |
| Shuai Jin  (2022) | Development only |  | Yes | Available for use at any time | |  |  | Low | Predictors were consistently defined and measured before diagnosis using routine clinical data; no subjective reassessment was performed. | |
| Wenbo Sheng  (2023) | Development only |  | Yes | Available for use at any time | |  |  | Low | Predictors were consistently defined and measured before diagnosis using routine clinical data; no subjective reassessment was performed. | |
| Sheng Pan  (2023) | Development only |  | Yes | Available for use at any time | |  |  | Low | Predictors were consistently defined and measured before diagnosis using routine clinical data; no subjective reassessment was performed. | |
| Xin Liu  (2024) | Development only |  | Yes | Available for use at any time | |  |  | Low | Predictors were consistently defined and measured before diagnosis using routine clinical data; no subjective reassessment was performed. | |

**Supplementary Table S1: Continued**

| Author (year) | Study type |  | 2.3 Are all predictors available at the time the model is intended to be used? | |  |  |  | Domain-level  RoB judgement | |  | |
| --- | --- | --- | --- | --- | --- | --- | --- | --- | --- | --- | --- |
|  |  |  | 2.3 | Notes 2.3 | |  |  | Reviewer's Judgement | RoB note | |  |
| Shengling Ma  (2024) | Development with external validation |  | Yes | Available for use at any time | |  |  | Low | Predictors were consistently defined and measured before diagnosis using routine clinical data; no subjective reassessment was performed. | |  |
| Lingling Liu  (2024) | Development only |  | Yes | Available for use at any time | |  |  | Low | Predictors were consistently defined and measured before diagnosis using routine clinical data; no subjective reassessment was performed. | |  |
| Dongcheng Shi  (2024) | Development only |  | Yes | Available for use at any time | |  |  | Low | Predictors were consistently defined and measured before diagnosis using routine clinical data; no subjective reassessment was performed. | |  |
| Conghui Wei  (2024) | Development only |  | Yes | Available for use at any time | |  |  | Low | Predictors were consistently defined and measured before diagnosis using routine clinical data; no subjective reassessment was performed. | |  |
| Haiyan Zhou  (2024) | Development only |  | Yes | Available for use at any time | |  |  | Low | Predictors were consistently defined and measured before diagnosis using routine clinical data; no subjective reassessment was performed. | |  |
| Jiangtao Ma  (2024) | Development only |  | Yes | Available for use at any time | |  |  | Low | Predictors were consistently defined and measured before diagnosis using routine clinical data; no subjective reassessment was performed. | |  |
| Jie Jin  (2024) | Development only |  | Yes | Available for use at any time | |  |  | Low | Predictors were consistently defined and measured before diagnosis using routine clinical data; no subjective reassessment was performed. | |  |

| Author (year) | Study type |  | 2.3 Are all predictors available at the time the model is intended to be used? | |  |  |  | Domain-level  RoB judgement | |  | |
| --- | --- | --- | --- | --- | --- | --- | --- | --- | --- | --- | --- |
|  |  |  | 2.3 | Notes 2.3 | |  |  | Reviewer's Judgement | RoB note | |  |
| Tao Yang  (2025) | Development only |  | Yes | Available for use at any time | |  |  | Low | Predictors were consistently defined and measured before diagnosis using routine clinical data; no subjective reassessment was performed. | |  |
| Kaibin Liu  (2025) | Development only |  | Yes | Available for use at any time | |  |  | Low | Predictors were consistently defined and measured before diagnosis using routine clinical data; no subjective reassessment was performed. | |  |
| Azadeh Tabari  (2025) | Development with external validation |  | Yes | Available for use at any time | |  |  | High | Predictors are not predefined but are selected in a data-driven manner during the modelling process. | |  |

**Supplementary Table S1: Continued**

**Supplementary Table S1: Continued**

| Author (year) | Study type |  | Domain-level applicability judgement | |  |
| --- | --- | --- | --- | --- | --- |
|  |  |  | Applicability | App note | |
| Li Wang  (2021) | Development only |  | Low concerns | All predictors are routinely collected in diagnostic settings. | |
| Logan Ryan  (2021) | Development only |  | Low concerns | All predictors are routinely collected in diagnostic settings. | |
| Rongqing Li  (2022) | Development only |  | Low concerns | All predictors are routinely collected in diagnostic settings. | |
| Shuai Jin  (2022) | Development only |  | High concerns | Routine testing is difficult to obtain. | |
| Wenbo Sheng  (2023) | Development only |  | Low concerns | All predictors are routinely collected in diagnostic settings. | |
| Sheng Pan  (2023) | Development only |  | Low concerns | All predictors are routinely collected in diagnostic settings. | |
| Xin Liu  (2024) | Development only |  | Low concerns | All predictors are routinely collected in diagnostic settings. | |

**Supplementary Table S1: Continued**

| Author (year) | Study type |  | Domain-level applicability judgement | |  |
| --- | --- | --- | --- | --- | --- |
|  |  |  | Applicability | App note | |
| Shengling Ma  (2024) | Development with external validation |  | Low concerns | All predictors are routinely collected in diagnostic settings. | |
| Lingling Liu  (2024) | Development only |  | Low concerns | All predictors are routinely collected in diagnostic settings. | |
| Dongcheng Shi  (2024) | Development only |  | High concerns | The studies included show that the timing of measurement cannot be routinely obtained. | |
| Conghui Wei  (2024) | Development only |  | Low concerns | All predictors are routinely collected in diagnostic settings. | |
| Haiyan Zhou  (2024) | Development only |  | High concerns | Routine testing is difficult to obtain. | |
| Jiangtao Ma  (2024) | Development only |  | High concerns | The studies included show that the timing of measurement cannot be routinely obtained. | |
| Jie Jin  (2024) | Development only |  | Low concerns | All predictors are routinely collected in diagnostic settings. | |

**Supplementary Table S1: Continued**

| Author (year) | Study type |  | Domain-level applicability judgement | |  |
| --- | --- | --- | --- | --- | --- |
|  |  |  | Applicability | App note | |
| Tao Yang  (2025) | Development only |  | Low concerns | All predictors are routinely collected in diagnostic settings. | |
| Kaibin Liu  (2025) | Development only |  | Low concerns | All predictors are routinely collected in diagnostic settings. | |
| Azadeh Tabari  (2025) | Development with external validation |  | Low concerns | All predictors are routinely collected in diagnostic settings. | |

**Supplementary Table S1: Continued**

| Author (year) | Study type |  | 3.1 was the outcome determined appropriately? | |  |  |  | 3.2 Was a pre-specified or standard outcome definition used? | |  |
| --- | --- | --- | --- | --- | --- | --- | --- | --- | --- | --- |
|  |  |  | 3.1 | Notes 3.1 | |  |  | 3.2 | Notes 3.2 | |
| Li Wang  (2021) | Development only |  | Yes | Outcome determined by gold standard | |  |  | Yes | Outcome definition followed established diagnostic criteria. | |
| Logan Ryan  (2021) | Development only |  | Yes | Outcome determined by gold standard | |  |  | Yes | Outcome definition followed established diagnostic criteria. | |
| Rongqing Li (2022) | Development only |  | Yes | Outcome determined by gold standard | |  |  | Yes | Outcome definition followed established diagnostic criteria. | |
| Shuai Jin  (2022) | Development only |  | Yes | Outcome determined by gold standard | |  |  | Yes | Outcome definition followed established diagnostic criteria. | |
| Wenbo Sheng  (2023) | Development only |  | Yes | Outcome determined by gold standard | |  |  | Yes | Outcome definition followed established diagnostic criteria. | |
| Sheng Pan  (2023) | Development only |  | Yes | Outcome determined by gold standard | |  |  | Yes | Outcome definition followed established diagnostic criteria. | |
| Xin Liu  (2024) | Development only |  | Yes | Outcome determined by gold standard | |  |  | Yes | Outcome definition followed established diagnostic criteria. | |

**Supplementary Table S1: Continued**

| Author (year) | Study type |  | 3.1 was the outcome determined appropriately? | |  |  |  | 3.2 Was a pre-specified or standard outcome definition used? | |  | |
| --- | --- | --- | --- | --- | --- | --- | --- | --- | --- | --- | --- |
|  |  |  | 3.1 | Notes 3.1 | |  |  | 3.2 | Notes 3.2 | |  |
| Shengling Ma  (2024) | Development with external validation |  | Yes | Outcome determined by gold standard | |  |  | Yes | Outcome definition followed established diagnostic criteria. | |  |
| Lingling Liu  (2024) | Development only |  | Yes | Outcome determined by gold standard | |  |  | Yes | Outcome definition followed established diagnostic criteria. | |  |
| Dongcheng Shi  (2024) | Development only |  | Yes | Outcome determined by gold standard | |  |  | Yes | Outcome definition followed established diagnostic criteria. | |  |
| Conghui Wei  (2024) | Development only |  | Yes | Outcome determined by gold standard | |  |  | Yes | Outcome definition followed established diagnostic criteria. | |  |
| Haiyan Zhou  (2024) | Development only |  | No | Measurement methods include non-gold standard D-dimer | |  |  | Yes | Outcome definition followed established diagnostic criteria. | |  |
| Jiangtao Ma  (2024) | Development only |  | Yes | Outcome determined by gold standard | |  |  | Yes | Outcome definition followed established diagnostic criteria. | |  |
| Jie Jin  (2024) | Development only |  | Yes | Outcome determined by gold standard | |  |  | Yes | Outcome definition followed established diagnostic criteria. | |  |

**Supplementary Table S1: Continued**

| Author (year) | Study type |  | 3.1 was the outcome determined appropriately? | |  |  |  | 3.2 Was a pre-specified or standard outcome definition used? | |  | |
| --- | --- | --- | --- | --- | --- | --- | --- | --- | --- | --- | --- |
|  |  |  | 3.1 | Notes 3.1 | |  |  | 3.2 | Notes 3.2 | |  |
| Tao Yang  (2025) | Development only |  | Yes | Outcome determined by gold standard | |  |  | Yes | Outcome definition followed established diagnostic criteria. | |  |
| Kaibin Liu  (2025) | Development only |  | Yes | Outcome determined by gold standard | |  |  | Yes | Outcome definition followed established diagnostic criteria. | |  |
| Azadeh Tabari  (2025) | Development with external validation |  | Yes | Outcome determined by gold standard | |  |  | Yes | Outcome definition followed established diagnostic criteria. | |  |

**Supplementary Table S1: Continued**

| Author (year) | Study type |  | 3.3 Were predictors excluded from the outcome definition? | |  |  |  | 3.4 Was the outcome defined and determined in a similar way for all participants? | |  |
| --- | --- | --- | --- | --- | --- | --- | --- | --- | --- | --- |
|  |  |  | 3.3 | Notes 3.3 | |  |  | 3.4 | Notes 3.4 | |
| Li Wang  (2021) | Development only |  | Yes | Predictors were independent from the outcome definition. | |  |  | Yes | All participants assessed using the same diagnostic procedure. | |
| Logan Ryan  (2021) | Development only |  | Yes | Predictors were independent from the outcome definition. | |  |  | Yes | All participants assessed using the same diagnostic procedure. | |
| Rongqing Li (2022) | Development only |  | Yes | Predictors were independent from the outcome definition. | |  |  | Yes | All participants assessed using the same diagnostic procedure. | |
| Shuai Jin  (2022) | Development only |  | Yes | Predictors were independent from the outcome definition. | |  |  | Yes | All participants assessed using the same diagnostic procedure. | |
| Wenbo Sheng  (2023) | Development only |  | Yes | Predictors were independent from the outcome definition. | |  |  | Yes | All participants assessed using the same diagnostic procedure. | |
| Sheng Pan  (2023) | Development only |  | Yes | Predictors were independent from the outcome definition. | |  |  | Yes | All participants assessed using the same diagnostic procedure. | |
| Xin Liu  (2024) | Development only |  | Yes | Predictors were independent from the outcome definition. | |  |  | Yes | All participants assessed using the same diagnostic procedure. | |

**Supplementary Table S1: Continued**

| Author (year) | Study type |  | 3.3 Were predictors excluded from the outcome definition? | |  |  |  | 3.4 Was the outcome defined and determined in a similar way for all participants? | |  | |
| --- | --- | --- | --- | --- | --- | --- | --- | --- | --- | --- | --- |
|  |  |  | 3.3 | Notes 3.3 | |  |  | 3.4 | Notes 3.4 | |  |
| Shengling Ma  (2024) | Development with external validation |  | Yes | Predictors were independent from the outcome definition. | |  |  | Yes | All participants assessed using the same diagnostic procedure. | |  |
| Lingling Liu  (2024) | Development only |  | Yes | Predictors were independent from the outcome definition. | |  |  | Yes | All participants assessed using the same diagnostic procedure. | |  |
| Dongcheng Shi  (2024) | Development only |  | Yes | Predictors were independent from the outcome definition. | |  |  | Yes | All participants assessed using the same diagnostic procedure. | |  |
| Conghui Wei  (2024) | Development only |  | Yes | Predictors were independent from the outcome definition. | |  |  | Yes | All participants assessed using the same diagnostic procedure. | |  |
| Haiyan Zhou  (2024) | Development only |  | No | Outcome definition excludes predictive factors | |  |  | Yes | All participants assessed using the same diagnostic procedure. | |  |
| Jiangtao Ma  (2024) | Development only |  | Yes | Predictors were independent from the outcome definition. | |  |  | Yes | All participants assessed using the same diagnostic procedure. | |  |
| Jie Jin  (2024) | Development only |  | Yes | Predictors were independent from the outcome definition. | |  |  | Yes | All participants assessed using the same diagnostic procedure. | |  |

**Supplementary Table S1: Continued**

| Author (year) | Study type |  | 3.3 Were predictors excluded from the outcome definition? | |  |  |  | 3.4 Was the outcome defined and determined in a similar way for all participants? | |  | |
| --- | --- | --- | --- | --- | --- | --- | --- | --- | --- | --- | --- |
|  |  |  | 3.3 | Notes 3.3 | |  |  | 3.4 | Notes 3.4 | |  |
| Tao Yang  (2025) | Development only |  | Yes | Predictors were independent from the outcome definition. | |  |  | Yes | All participants assessed using the same diagnostic procedure. | |  |
| Kaibin Liu  (2025) | Development only |  | Yes | Predictors were independent from the outcome definition. | |  |  | Yes | All participants assessed using the same diagnostic procedure. | |  |
| Azadeh Tabari  (2025) | Development with external validation |  | No | The researchers did not distinguish between the sources of the predictors and the outcome variables. | |  |  | Yes | All participants assessed using the same diagnostic procedure. | |  |

**Supplementary Table S1: Continued**

| Author (year) | Study type |  | 3.5 Was the outcome determine without knowledge of predictor information? | |  |  |  | 3.6 Was the time interval between predictor assessment and outcome determination appropriate? | |  |
| --- | --- | --- | --- | --- | --- | --- | --- | --- | --- | --- |
|  |  |  | 3.5 | Notes 3.5 | |  |  | 3.6 | Notes 3.6 | |
| Li Wang  (2021) | Development only |  | Yes | Outcome determined independently and blinded to predictor data. | |  |  | Yes | Predictors and outcome assessed at the same clinical encounter. | |
| Logan Ryan  (2021) | Development only |  | Yes | Outcome determined independently and blinded to predictor data. | |  |  | Yes | Predictors and outcome assessed at the same clinical encounter. | |
| Rongqing Li (2022) | Development only |  | Yes | Outcome determined independently and blinded to predictor data. | |  |  | Yes | Predictors and outcome assessed at the same clinical encounter. | |
| Shuai Jin  (2022) | Development only |  | Yes | Outcome determined independently and blinded to predictor data. | |  |  | Yes | Predictors and outcome assessed at the same clinical encounter. | |
| Wenbo Sheng  (2023) | Development only |  | Yes | Outcome determined independently and blinded to predictor data. | |  |  | Yes | Predictors and outcome assessed at the same clinical encounter. | |
| Sheng Pan  (2023) | Development only |  | Yes | Outcome determined independently and blinded to predictor data. | |  |  | Yes | Predictors and outcome assessed at the same clinical encounter. | |
| Xin Liu  (2024) | Development only |  | Yes | Outcome determined independently and blinded to predictor data. | |  |  | Yes | Predictors and outcome assessed at the same clinical encounter. | |

**Supplementary Table S1: Continued**

| Author (year) | Study type |  | 3.5 Was the outcome determine without knowledge of predictor information? | |  |  |  | 3.6 Was the time interval between predictor assessment and outcome determination appropriate? | |  | |
| --- | --- | --- | --- | --- | --- | --- | --- | --- | --- | --- | --- |
|  |  |  | 3.5 | Notes 3.5 | |  |  | 3.6 | Notes 3.6 | |  |
| Shengling Ma  (2024) | Development with external validation |  | Yes | Outcome determined independently and blinded to predictor data. | |  |  | Yes | Predictors and outcome assessed at the same clinical encounter. | |  |
| Lingling Liu  (2024) | Development only |  | Yes | Outcome determined independently and blinded to predictor data. | |  |  | Yes | Predictors and outcome assessed at the same clinical encounter. | |  |
| Dongcheng Shi  (2024) | Development only |  | Yes | Outcome determined independently and blinded to predictor data. | |  |  | Yes | Predictors and outcome assessed at the same clinical encounter. | |  |
| Conghui Wei  (2024) | Development only |  | Yes | Outcome determined independently and blinded to predictor data. | |  |  | Yes | Predictors and outcome assessed at the same clinical encounter. | |  |
| Haiyan Zhou  (2024) | Development only |  | No | The outcome is determined by predictive factors. | |  |  | Yes | Predictors and outcome assessed at the same clinical encounter. | |  |
| Jiangtao Ma  (2024) | Development only |  | Yes | Outcome determined independently and blinded to predictor data. | |  |  | Yes | Predictors and outcome assessed at the same clinical encounter. | |  |
| Jie Jin  (2024) | Development only |  | Yes | Outcome determined independently and blinded to predictor data. | |  |  | Yes | Predictors and outcome assessed at the same clinical encounter. | |  |

**Supplementary Table S1: Continued**

| Author (year) | Study type |  | 3.5 Was the outcome determine without knowledge of predictor information? | |  |  |  | 3.6 Was the time interval between predictor assessment and outcome determination appropriate? | |  | |
| --- | --- | --- | --- | --- | --- | --- | --- | --- | --- | --- | --- |
|  |  |  | 3.5 | Notes 3.5 | |  |  | 3.6 | Notes 3.6 | |  |
| Tao Yang  (2025) | Development only |  | Yes | Outcome determined independently and blinded to predictor data. | |  |  | Yes | Predictors and outcome assessed at the same clinical encounter. | |  |
| Kaibin Liu  (2025) | Development only |  | Yes | Outcome determined independently and blinded to predictor data. | |  |  | Yes | Predictors and outcome assessed at the same clinical encounter. | |  |
| Azadeh Tabari  (2025) | Development with external validation |  | Yes | Outcome determined independently and blinded to predictor data. | |  |  | Yes | Predictors and outcome assessed at the same clinical encounter. | |  |

**Supplementary Table S1: Continued**

| Author (year) | Study type |  | Domain-level  RoB judgement | |  |
| --- | --- | --- | --- | --- | --- |
|  |  |  | Reviewer's Judgement | RoB note | |
| Li Wang  (2021) | Development only |  | Low | Outcomes were defined using standardized diagnostic criteria, assessed identically for all participants, and determined independently of predictors. | |
| Logan Ryan  (2021) | Development only |  | Low | Outcomes were defined using standardized diagnostic criteria, assessed identically for all participants, and determined independently of predictors. | |
| Rongqing Li (2022) | Development only |  | Low | Outcomes were defined using standardized diagnostic criteria, assessed identically for all participants, and determined independently of predictors. | |
| Shuai Jin  (2022) | Development only |  | Low | Outcomes were defined using standardized diagnostic criteria, assessed identically for all participants, and determined independently of predictors. | |
| Wenbo Sheng  (2023) | Development only |  | Low | Outcomes were defined using standardized diagnostic criteria, assessed identically for all participants, and determined independently of predictors. | |
| Sheng Pan  (2023) | Development only |  | Low | Outcomes were defined using standardized diagnostic criteria, assessed identically for all participants, and determined independently of predictors. | |
| Xin Liu  (2024) | Development only |  | Low | Outcomes were defined using standardized diagnostic criteria, assessed identically for all participants, and determined independently of predictors. | |

**Supplementary Table S1: Continued**

| Author (year) | Study type |  | Domain-level  RoB judgement | |  |
| --- | --- | --- | --- | --- | --- |
|  |  |  | RoB judgement | RoB note | |
| Shengling Ma  (2024) | Development with external validation |  | Low | Outcomes were defined using standardized diagnostic criteria, assessed identically for all participants, and determined independently of predictors. | |
| Lingling Liu  (2024) | Development only |  | Low | Outcomes were defined using standardized diagnostic criteria, assessed identically for all participants, and determined independently of predictors. | |
| Dongcheng Shi  (2024) | Development only |  | Low | Outcomes were defined using standardized diagnostic criteria, assessed identically for all participants, and determined independently of predictors. | |
| Conghui Wei  (2024) | Development only |  | Low | Outcomes were defined using standardized diagnostic criteria, assessed identically for all participants, and determined independently of predictors. | |
| Haiyan Zhou  (2024) | Development only |  | High | Outcomes incorporated into non-gold standard | |
| Jiangtao Ma  (2024) | Development only |  | Low | Outcomes were defined using standardized diagnostic criteria, assessed identically for all participants, and determined independently of predictors. | |
| Jie Jin  (2024) | Development only |  | Low | Outcomes were defined using standardized diagnostic criteria, assessed identically for all participants, and determined independently of predictors. | |

**Supplementary Table S1: Continued**

| Author (year) | Study type |  | Domain-level  RoB judgement | |  |
| --- | --- | --- | --- | --- | --- |
|  |  |  | RoB judgement | RoB note | |
| Tao Yang  (2025) | Development only |  | Low | Outcomes were defined using standardized diagnostic criteria, assessed identically for all participants, and determined independently of predictors. | |
| Kaibin Liu  (2025) | Development only |  | Low | Outcomes were defined using standardized diagnostic criteria, assessed identically for all participants, and determined independently of predictors. | |
| Azadeh Tabari  (2025) | Development with external validation |  | High | Feature selection was not performed. | |

**Supplementary Table S1: Continued**

| Author (year) | Study type |  | Domain-level applicability judgement | |  |
| --- | --- | --- | --- | --- | --- |
|  |  |  | Applicability | App note | |
| Li Wang  (2021) | Development only |  | Low concerns | Outcome reflects real-world diagnostic endpoint consistent with clinical use. | |
| Logan Ryan  (2021) | Development only |  | Low concerns | Outcome reflects real-world diagnostic endpoint consistent with clinical use. | |
| Rongqing Li (2022) | Development only |  | Low concerns | Outcome reflects real-world diagnostic endpoint consistent with clinical use. | |
| Shuai Jin  (2022) | Development only |  | Low concerns | Outcome reflects real-world diagnostic endpoint consistent with clinical use. | |
| Wenbo Sheng  (2023) | Development only |  | Low concerns | Outcome reflects real-world diagnostic endpoint consistent with clinical use. | |
| Sheng Pan  (2023) | Development only |  | Low concerns | Outcome reflects real-world diagnostic endpoint consistent with clinical use. | |
| Xin Liu  (2024) | Development only |  | Low concerns | Outcome reflects real-world diagnostic endpoint consistent with clinical use. | |

**Supplementary Table S1: Continued**

| Author (year) | Study type |  | Domain-level applicability judgement | |  |
| --- | --- | --- | --- | --- | --- |
|  |  |  | Applicability | App note | |
| Shengling Ma  (2024) | Development with external validation |  | Low concerns | Outcome reflects real-world diagnostic endpoint consistent with clinical use. | |
| Lingling Liu  (2024) | Development only |  | Low concerns | Outcome reflects real-world diagnostic endpoint consistent with clinical use. | |
| Dongcheng Shi  (2024) | Development only |  | Low concerns | Outcome reflects real-world diagnostic endpoint consistent with clinical use. | |
| Conghui Wei  (2024) | Development only |  | Low concerns | Outcome reflects real-world diagnostic endpoint consistent with clinical use. | |
| Haiyan Zhou  (2024) | Development only |  | Low concerns | Outcome reflects real-world diagnostic endpoint consistent with clinical use. | |
| Jiangtao Ma  (2024) | Development only |  | Low concerns | Outcome reflects real-world diagnostic endpoint consistent with clinical use. | |
| Jie Jin  (2024) | Development only |  | High concerns | The outcome includes both deep vein thrombosis and pulmonary embolism. | |

**Supplementary Table S1: Continued**

| Author (year) | Study type |  | Domain-level applicability judgement | |  |
| --- | --- | --- | --- | --- | --- |
|  |  |  | Applicability | App note | |
| Tao Yang  (2025) | Development only |  | Low concerns | Outcome reflects real-world diagnostic endpoint consistent with clinical use. | |
| Kaibin Liu  (2025) | Development only |  | Low concerns | Outcome reflects real-world diagnostic endpoint consistent with clinical use. | |
| Azadeh Tabari  (2025) | Development with external validation |  | Low concerns | Outcome reflects real-world diagnostic endpoint consistent with clinical use. | |

**Supplementary Table S1: Continued**

| Author (year) | Study type |  | 4.1 Were there a reasonable number of participants with the outcome? | |  |  |  | 4.2 Were continuous and categorical predictors handled appropriately? | |  |
| --- | --- | --- | --- | --- | --- | --- | --- | --- | --- | --- |
|  |  |  | 4.1 | Notes 4.1 | |  |  | 4.2 | Notes 4.2 | |
| Li Wang  (2021) | Development only |  | Yes | Sample size adequate, with sufficient events per variable | |  |  | No information | No mention of variable handling methods | |
| Logan Ryan  (2021) | Development only |  | Yes | Sample size adequate, with sufficient events per variable | |  |  | Yes | Variable handling is appropriate | |
| Rongqing Li (2022) | Development only |  | Yes | Sample size adequate, with sufficient events per variable | |  |  | Yes | Variable handling is appropriate | |
| Shuai Jin  (2022) | Development only |  | Yes | Sample size adequate, with sufficient events per variable | |  |  | Yes | Variable handling is appropriate | |
| Wenbo Sheng  (2023) | Development only |  | Yes | Sample size adequate, with sufficient events per variable | |  |  | Yes | Variable handling is appropriate | |
| Sheng Pan  (2023) | Development only |  | Yes | Sample size adequate, with sufficient events per variable | |  |  | No information | No mention of variable handling methods | |
| Xin Liu  (2024) | Development only |  | Yes | Sample size adequate, with sufficient events per variable | |  |  | Yes | Variable handling is appropriate | |

**Supplementary Table S1: Continued**

| Author (year) | Study type |  | 4.1 Were there a reasonable number of participants with the outcome? | |  |  |  | 4.2 Were continuous and categorical predictors handled appropriately? | |  | |
| --- | --- | --- | --- | --- | --- | --- | --- | --- | --- | --- | --- |
|  |  |  | 4.1 | Notes 4.1 | |  |  | 4.2 | Notes 4.2 | |  |
| Shengling Ma  (2024) | Development with external validation |  | Yes | Sample size adequate, with sufficient events per variable | |  |  | No information | No mention of variable handling methods | |  |
| Lingling Liu  (2024) | Development only |  | Yes | Sample size adequate, with sufficient events per variable | |  |  | Yes | Variable handling is appropriate | |  |
| Dongcheng Shi  (2024) | Development only |  | Yes | Sample size adequate, with sufficient events per variable | |  |  | No information | No mention of variable handling methods | |  |
| Conghui Wei  (2024) | Development only |  | Yes | Sample size adequate, with sufficient events per variable | |  |  | Yes | Variable handling is appropriate | |  |
| Haiyan Zhou  (2024) | Development only |  | No | Inadequate number of outcome events, risk of overfitting. | |  |  | Yes | Variable handling is appropriate | |  |
| Jiangtao Ma  (2024) | Development only |  | Yes | Sample size adequate, with sufficient events per variable | |  |  | Yes | Variable handling is appropriate | |  |
| Jie Jin  (2024) | Development only |  | Yes | Sample size adequate, with sufficient events per variable | |  |  | Yes | Variable handling is appropriate | |  |

**Supplementary Table S1: Continued**

| Author (year) | Study type |  | 4.1 Were there a reasonable number of participants with the outcome? | |  |  |  | 4.2 Were continuous and categorical predictors handled appropriately? | |  | |
| --- | --- | --- | --- | --- | --- | --- | --- | --- | --- | --- | --- |
|  |  |  | 4.1 | Notes 4.1 | |  |  | 4.2 | Notes 4.2 | |  |
| Tao Yang  (2025) | Development only |  | Yes | Sample size adequate, with sufficient events per variable | |  |  | No information | No mention of variable handling methods | |  |
| Kaibin Liu  (2025) | Development only |  | Yes | Sample size adequate, with sufficient events per variable | |  |  | No information | No mention of variable handling methods | |  |
| Azadeh Tabari  (2025) | Development with external validation |  | Yes | Sample size adequate, with sufficient events per variable | |  |  | Yes | Variable handling is appropriate | |  |

**Supplementary Table S1: Continued**

| Author (year) | Study type |  | 4.3 Were all enrolled participants included in the analysis? | |  |  |  | 4.4 Were participants with missing data handled appropriately? | |  |
| --- | --- | --- | --- | --- | --- | --- | --- | --- | --- | --- |
|  |  |  | 4.3 | Notes 4.3 | |  |  | 4.4 | Notes 4.4 | |
| Li Wang  (2021) | Development only |  | Yes | All eligible participants included in the final model analysis. | |  |  | No information | Missing data handling not mentioned | |
| Logan Ryan  (2021) | Development only |  | Yes | All eligible participants included in the final model analysis. | |  |  | No information | Missing data handling not mentioned | |
| Rongqing Li (2022) | Development only |  | Yes | All eligible participants included in the final model analysis. | |  |  | No information | Missing data handling not mentioned | |
| Shuai Jin  (2022) | Development only |  | Yes | All eligible participants included in the final model analysis. | |  |  | Yes | Missing data has been processed. | |
| Wenbo Sheng  (2023) | Development only |  | Yes | All eligible participants included in the final model analysis. | |  |  | Yes | Missing data has been processed. | |
| Sheng Pan  (2023) | Development only |  | Yes | All eligible participants included in the final model analysis. | |  |  | No | Inadequate handling of missing data | |
| Xin Liu  (2024) | Development only |  | Yes | All eligible participants included in the final model analysis. | |  |  | Yes | Missing data has been processed. | |

**Supplementary Table S1: Continued**

| Author (year) | Study type |  | 4.3 Were all enrolled participants included in the analysis? | |  |  |  | 4.4 Were participants with missing data handled appropriately? | |  | |
| --- | --- | --- | --- | --- | --- | --- | --- | --- | --- | --- | --- |
|  |  |  | 4.3 | Notes 4.3 | |  |  | 4.4 | Notes 4.4 | |  |
| Shengling Ma  (2024) | Development with external validation |  | Yes | All eligible participants included in the final model analysis. | |  |  | Yes | Missing data has been processed. | |  |
| Lingling Liu  (2024) | Development only |  | Yes | All eligible participants included in the final model analysis. | |  |  | Yes | Missing data has been processed. | |  |
| Dongcheng Shi  (2024) | Development only |  | Yes | All eligible participants included in the final model analysis. | |  |  | No information | Missing data handling not mentioned | |  |
| Conghui Wei  (2024) | Development only |  | Yes | All eligible participants included in the final model analysis. | |  |  | Yes | Missing data has been processed. | |  |
| Haiyan Zhou  (2024) | Development only |  | Yes | All eligible participants included in the final model analysis. | |  |  | No information | Missing data handling not mentioned | |  |
| Jiangtao Ma  (2024) | Development only |  | Yes | All eligible participants included in the final model analysis. | |  |  | No information | Missing data handling not mentioned | |  |
| Jie Jin  (2024) | Development only |  | Yes | All eligible participants included in the final model analysis. | |  |  | Yes | Missing data has been processed. | |  |

**Supplementary Table S1: Continued**

| Author (year) | Study type |  | 4.3 Were all enrolled participants included in the analysis? | |  |  |  | 4.4 Were participants with missing data handled appropriately? | |  | |
| --- | --- | --- | --- | --- | --- | --- | --- | --- | --- | --- | --- |
|  |  |  | 4.3 | Notes 4.3 | |  |  | 4.4 | Notes 4.4 | |  |
| Tao Yang  (2025) | Development only |  | Yes | All eligible participants included in the final model analysis. | |  |  | No information | Missing data handling not mentioned | |  |
| Kaibin Liu  (2025) | Development only |  | Yes | All eligible participants included in the final model analysis. | |  |  | No information | Missing data handling not mentioned | |  |
| Azadeh Tabari  (2025) | Development with external validation |  | Yes | All eligible participants included in the final model analysis. | |  |  | Yes | Missing data has been processed. | |  |

**Supplementary Table S1: Continued**

| Author (year) | Study type |  | 4.5 Was selection of predictors based on univariable analysis avoided? | |  |  |  | 4.6 Were complexities in the data (e.g., censoring, competing risks, sampling of control participants) accounted for appropriately? | |  |
| --- | --- | --- | --- | --- | --- | --- | --- | --- | --- | --- |
|  |  |  | 4.5 | Notes 4.5 | |  |  | 4.6 | Notes 4.6 | |
| Li Wang  (2021) | Development only |  | Yes | Variable selection was not limited to univariate analysis. | |  |  | Yes | dealt with appropriately | |
| Logan Ryan  (2021) | Development only |  | Yes | Variable selection was not limited to univariate analysis. | |  |  | Yes | dealt with appropriately | |
| Rongqing Li (2022) | Development only |  | Yes | Variable selection was not limited to univariate analysis. | |  |  | Yes | dealt with appropriately | |
| Shuai Jin  (2022) | Development only |  | Yes | Variable selection was not limited to univariate analysis. | |  |  | Yes | dealt with appropriately | |
| Wenbo Sheng  (2023) | Development only |  | Yes | Variable selection was not limited to univariate analysis. | |  |  | Yes | dealt with appropriately | |
| Sheng Pan  (2023) | Development only |  | Yes | Variable selection was not limited to univariate analysis. | |  |  | Yes | dealt with appropriately | |
| Xin Liu  (2024) | Development only |  | Yes | Variable selection was not limited to univariate analysis. | |  |  | Yes | dealt with appropriately | |

**Supplementary Table S1: Continued**

| Author (year) | Study type |  | 4.5 Was selection of predictors based on univariable analysis avoided? | |  |  |  | 4.6 Were complexities in the data (e.g., censoring, competing risks, sampling of control participants) accounted for appropriately? | |  | |
| --- | --- | --- | --- | --- | --- | --- | --- | --- | --- | --- | --- |
|  |  |  | 4.5 | Notes 4.5 | |  |  | 4.6 | Notes 4.6 | |  |
| Shengling Ma  (2024) | Development with external validation |  | Yes | Variable selection was not limited to univariate analysis. | |  |  | Yes | dealt with appropriately | |  |
| Lingling Liu  (2024) | Development only |  | Yes | Variable selection was not limited to univariate analysis. | |  |  | Yes | dealt with appropriately | |  |
| Dongcheng Shi  (2024) | Development only |  | Yes | Variable selection was not limited to univariate analysis. | |  |  | Yes | dealt with appropriately | |  |
| Conghui Wei  (2024) | Development only |  | Yes | Variable selection was not limited to univariate analysis. | |  |  | Yes | dealt with appropriately | |  |
| Haiyan Zhou  (2024) | Development only |  | Yes | Variable selection was not limited to univariate analysis. | |  |  | Yes | dealt with appropriately | |  |
| Jiangtao Ma  (2024) | Development only |  | Yes | Variable selection was not limited to univariate analysis. | |  |  | Yes | dealt with appropriately | |  |
| Jie Jin  (2024) | Development only |  | Yes | Variable selection was not limited to univariate analysis. | |  |  | Yes | dealt with appropriately | |  |

**Supplementary Table S1: Continued**

| Author (year) | Study type |  | 4.5 Was selection of predictors based on univariable analysis avoided? | |  |  |  | 4.6 Were complexities in the data (e.g., censoring, competing risks, sampling of control participants) accounted for appropriately? | |  | |
| --- | --- | --- | --- | --- | --- | --- | --- | --- | --- | --- | --- |
|  |  |  | 4.5 | Notes 4.5 | |  |  | 4.6 | Notes 4.6 | |  |
| Tao Yang  (2025) | Development only |  | Yes | Variable selection was not limited to univariate analysis. | |  |  | Yes | dealt with appropriately | |  |
| Kaibin Liu  (2025) | Development only |  | Yes | Variable selection was not limited to univariate analysis. | |  |  | Yes | dealt with appropriately | |  |
| Azadeh Tabari  (2025) | Development with external validation |  | No | No feature selection was performed. | |  |  | Yes | dealt with appropriately | |  |

**Supplementary Table S1: Continued**

| Author (year) | Study type |  | 4.7 Were relevant model performance measures evaluated appropriately? | |  |  |  | 4.8 Were model overfitting and optimism in model performance accounted for? | |  |
| --- | --- | --- | --- | --- | --- | --- | --- | --- | --- | --- |
|  |  |  | 4.7 | Notes 4.7 | |  |  | 4.8 | Notes 4.8 | |
| Li Wang  (2021) | Development only |  | Yes | Possesses a comprehensive set of evaluation indicators | |  |  | Yes | Internal validation has been conducted. | |
| Logan Ryan  (2021) | Development only |  | No | Calibration curve not considered | |  |  | No | Consider model overfitting | |
| Rongqing Li (2022) | Development only |  | Yes | Possesses a comprehensive set of evaluation indicators | |  |  | Yes | Internal validation has been conducted. | |
| Shuai Jin  (2022) | Development only |  | Yes | Possesses a comprehensive set of evaluation indicators | |  |  | Yes | Internal validation has been conducted. | |
| Wenbo Sheng  (2023) | Development only |  | Yes | Possesses a comprehensive set of evaluation indicators | |  |  | Yes | Internal validation has been conducted. | |
| Sheng Pan  (2023) | Development only |  | Yes | Possesses a comprehensive set of evaluation indicators | |  |  | Yes | Internal validation has been conducted. | |
| Xin Liu  (2024) | Development only |  | Yes | Possesses a comprehensive set of evaluation indicators | |  |  | Yes | Internal validation has been conducted. | |

**Supplementary Table S1: Continued**

| Author (year) | Study type |  | 4.7 Were relevant model performance measures evaluated appropriately? | |  |  |  | 4.8 Were model overfitting and optimism in model performance accounted for? | |  | |
| --- | --- | --- | --- | --- | --- | --- | --- | --- | --- | --- | --- |
|  |  |  | 4.7 | Notes 4.7 | |  |  | 4.8 | Notes 4.8 | |  |
| Shengling Ma  (2024) | Development with external validation |  | Yes | Possesses a comprehensive set of evaluation indicators | |  |  | Yes | Internal validation has been conducted. | |  |
| Lingling Liu  (2024) | Development only |  | Yes | Possesses a comprehensive set of evaluation indicators | |  |  | Yes | Internal validation has been conducted. | |  |
| Dongcheng Shi  (2024) | Development only |  | Yes | Possesses a comprehensive set of evaluation indicators | |  |  | Yes | Internal validation has been conducted. | |  |
| Conghui Wei  (2024) | Development only |  | No | Calibration curve not considered | |  |  | No | Consider model overfitting | |  |
| Haiyan Zhou  (2024) | Development only |  | Yes | Possesses a comprehensive set of evaluation indicators | |  |  | Yes | Internal validation has been conducted. | |  |
| Jiangtao Ma  (2024) | Development only |  | Yes | Possesses a comprehensive set of evaluation indicators | |  |  | Yes | Internal validation has been conducted. | |  |
| Jie Jin  (2024) | Development only |  | Yes | Possesses a comprehensive set of evaluation indicators | |  |  | Yes | Internal validation has been conducted. | |  |

**Supplementary Table S1: Continued**

| Author (year) | Study type |  | 4.7 Were relevant model performance measures evaluated appropriately? | |  |  |  | 4.8 Were model overfitting and optimism in model performance accounted for? | |  | |
| --- | --- | --- | --- | --- | --- | --- | --- | --- | --- | --- | --- |
|  |  |  | 4.7 | Notes 4.7 | |  |  | 4.8 | Notes 4.8 | |  |
| Tao Yang  (2025) | Development only |  | Yes | Possesses a comprehensive set of evaluation indicators | |  |  | No | Consider model overfitting | |  |
| Kaibin Liu  (2025) | Development only |  | Yes | Possesses a comprehensive set of evaluation indicators | |  |  | Yes | Internal validation has been conducted. | |  |
| Azadeh Tabari  (2025) | Development with external validation |  | No | Calibration curve not considered | |  |  | Yes | Internal validation has been conducted. | |  |

**Supplementary Table S1: Continued**

| Author (year) | Study type |  | 4.9 Do predictors and their assigned weights in the final model correspond to the results from the reported multivariable analysis? | |  |  |  | Domain-level  RoB judgement | |  |
| --- | --- | --- | --- | --- | --- | --- | --- | --- | --- | --- |
|  |  |  | 4.9 | Notes 4.9 | |  |  | Reviewer's Judgement | RoB note | |
| Li Wang  (2021) | Development only |  | Yes | Predictors and coefficients consistent with final multivariable model. | |  |  | Unclear | No mention of missing data and variable handling | |
| Logan Ryan  (2021) | Development only |  | Yes | Predictors and coefficients consistent with final multivariable model. | |  |  | High | Variable omission and selection bias were not addressed, nor were calibration and model fit considered. | |
| Rongqing Li (2022) | Development only |  | Yes | Predictors and coefficients consistent with final multivariable model. | |  |  | Unclear | No mention of missing data handling | |
| Shuai Jin  (2022) | Development only |  | Yes | Predictors and coefficients consistent with final multivariable model. | |  |  | Low | Adequate sample size, appropriate handling of missing data and validation performed. | |
| Wenbo Sheng  (2023) | Development only |  | Yes | Predictors and coefficients consistent with final multivariable model. | |  |  | Low | Adequate sample size, appropriate handling of missing data and validation performed. | |
| Sheng Pan  (2023) | Development only |  | Yes | Predictors and coefficients consistent with final multivariable model. | |  |  | High | Inadequate handling of missing data | |
| Xin Liu  (2024) | Development only |  | Yes | Predictors and coefficients consistent with final multivariable model. | |  |  | Low | Adequate sample size, appropriate handling of missing data and validation performed. | |

**Supplementary Table S1: Continued**

| Author (year) | Study type |  | 4.9 Do predictors and their assigned weights in the final model correspond to the results from the reported multivariable analysis? | |  |  |  | Domain-level  RoB judgement | |  | |
| --- | --- | --- | --- | --- | --- | --- | --- | --- | --- | --- | --- |
|  |  |  | 4.9 | Notes 4.9 | |  |  | Reviewer's Judgement | RoB note | |  |
| Shengling Ma  (2024) | Development with external validation |  | Yes | Predictors and coefficients consistent with final multivariable model. | |  |  | Unclear | Variable handling not mentioned | |  |
| Lingling Liu  (2024) | Development only |  | Yes | Predictors and coefficients consistent with final multivariable model. | |  |  | Low | Adequate sample size, appropriate handling of missing data and validation performed. | |  |
| Dongcheng Shi  (2024) | Development only |  | Yes | Predictors and coefficients consistent with final multivariable model. | |  |  | Unclear | No mention of missing data and variable handling | |  |
| Conghui Wei  (2024) | Development only |  | Yes | Predictors and coefficients consistent with final multivariable model. | |  |  | High | Calibration methods and model fitting were not considered. | |  |
| Haiyan Zhou  (2024) | Development only |  | Yes | Predictors and coefficients consistent with final multivariable model. | |  |  | High | Insufficient positive events; no mention of missing data handling | |  |
| Jiangtao Ma  (2024) | Development only |  | Yes | Predictors and coefficients consistent with final multivariable model. | |  |  | Unclear | No mention of missing data handling | |  |
| Jie Jin  (2024) | Development only |  | Yes | Predictors and coefficients consistent with final multivariable model. | |  |  | Low | Adequate sample size, appropriate handling of missing data and validation performed. | |  |

**Supplementary Table S1: Continued**

| Author (year) | Study type |  | 4.9 Do predictors and their assigned weights in the final model correspond to the results from the reported multivariable analysis? | |  |  |  | Domain-level  RoB judgement | |  | |
| --- | --- | --- | --- | --- | --- | --- | --- | --- | --- | --- | --- |
|  |  |  | 4.9 | Notes 4.9 | |  |  | Reviewer's Judgement | RoB note | |  |
| Tao Yang  (2025) | Development only |  | Yes | Predictors and coefficients consistent with final multivariable model. | |  |  | High | Consideration of model overfitting and variable handling not mentioned | |  |
| Kaibin Liu  (2025) | Development only |  | Yes | Predictors and coefficients consistent with final multivariable model. | |  |  | Unclear | Variables and missing data handling were not mentioned. | |  |
| Azadeh Tabari  (2025) | Development with external validation |  | Yes | Predictors and coefficients consistent with final multivariable model. | |  |  | High | No consideration of calibration methods; no mention of variable screening. | |  |

**Supplementary Table S1: Continued**

| Author (year) | Study type |  | Overall judgement about RoB | Overall judgement about applicability |
| --- | --- | --- | --- | --- |
|  |  |  | Reviewer's Judgement | Reviewer's Judgement |
| Li Wang  (2021) | Development only |  | Unclear | Low concerns |
| Logan Ryan  (2021) | Development only |  | High | Low concerns |
| Rongqing Li  (2022) | Development only |  | Unclear | Low concerns |
| Shuai Jin  (2022) | Development only |  | Low | High concerns |
| Wenbo Sheng  (2023) | Development only |  | Low | Low concerns |
| Sheng Pan  (2023) | Development only |  | High | Low concerns |
| Xin Liu  (2024) | Development only |  | Low | Low concerns |
| Shengling Ma  (2024) | Development with external validation |  | Unclear | Low concerns |
| Lingling Liu  (2024) | Development only |  | Low | Low concerns |
| Dongcheng Shi  (2024) | Development only |  | Unclear | High concerns |
| Conghui Wei  (2024) | Development only |  | High | Low concerns |
| Haiyan Zhou  (2024) | Development only |  | High | High concerns |
| Jiangtao Ma  (2024) | Development only |  | Unclear | High concerns |
| Jie Jin  (2024) | Development only |  | Low | High concerns |
| Tao Yang  (2025) | Development only |  | High | Low concerns |
| Kaibin Liu  (2025) | Development only |  | Unclear | Low concerns |
| Azadeh Tabari  (2025) | Development with external validation |  | High | Low concerns |
